# EtcABC, a Putative EII Complex, Regulates Type 3 Fimbriae via CRP-cAMP Signaling in *Klebsiella pneumoniae*

**DOI:** 10.3389/fmicb.2019.01558

**Published:** 2019-07-09

**Authors:** Novaria Sari Dewi Panjaitan, Yu-Tze Horng, Shih-Wen Cheng, Wen-Ting Chung, Po-Chi Soo

**Affiliations:** ^1^Institute of Medical Sciences, College of Medicine, Tzu Chi University, Hualien City, Taiwan; ^2^Department of Laboratory Medicine and Biotechnology, College of Medicine, Tzu Chi University, Hualien City, Taiwan

**Keywords:** *Klebsiella pneumoniae*, carbohydrate phosphotransferase system, biofilm, type 3 fimbriae, CRP-cAMP

## Abstract

Biofilm formation by *Klebsiella pneumoniae* on indwelling medical devices increases the risk of infection. Both type 1 and type 3 fimbriae are important factors in biofilm formation by *K. pneumoniae*. We found that a putative enzyme II (EII) complex of the phosphoenolpyruvate (PEP):carbohydrate phosphotransferase system (PTS), *etcA* (EIIA)-*etcB* (EIIB)-*etcC* (EIIC), regulated biofilm and type 3 fimbriae formation by *K. pneumoniae* STU1. In this study, the regulatory mechanism of *etcABC* in *K. pneumoniae* type 3 fimbriae formation was investigated. We found via quantitative RT-PCR that overexpression of *etcABC* enhanced the transcription level of the *mrk* operon, which is involved in type 3 fimbriae synthesis, and reduced the transcription level of the *fim* operon, which is involved in type 1 fimbriae synthesis. To gain further insight into the role of *etcABC* in type 3 fimbriae synthesis, we analyzed the region upstream of the *mrk* operon and found the potential cyclic 3′5′-adenosine monophosphate (cAMP) receptor protein (CRP) binding site. After *crp* was deleted in *K. pneumoniae* STU1 and two clinical isolates, these three *crp* mutant strains could not express MrkA, the major subunit of the fimbrial shaft, indicating that CRP positively regulated type 3 fimbriae synthesis. Moreover, a *crp* mutant overexpressing *etcABC* could not express MrkA, indicating that the regulation of type 3 fimbriae by *etcABC* was dependent on CRP. In addition, deletion of *cyaA*, which encodes the adenylyl cyclase that synthesizes cAMP, and deletion of *crr*, which encodes the glucose-specific EIIA, led to a reduction in *lac* operon regulation and therefore bacterial lactose uptake in *K. pneumoniae*. Exogenous cAMP but not *etcABC* overexpression compensated for the role of *cyaA* in bacterial lactose uptake. However, either *etcABC* overexpression or exogenous cAMP compensated for the role of *crr* in bacterial *lac* operon regulation that would eventually restore lactose uptake. We also found via ELISA and the *luxCDABE* reporter system that overexpression of *etcABC* increased intracellular cAMP levels and the transcription level of *crp*, respectively, in *K. pneumoniae*. In conclusion, overexpression of *etcABC* positively regulated cAMP production and cAMP-CRP activity to activate the *mrk* operon, resulting in increased type 3 fimbriae synthesis in *K. pneumoniae*.

## Introduction

Patients using medical devices easily become vulnerable to microbial infection. Biofilm formation on medical device surfaces increases the risk of infection. A 10-year survey of healthcare-associated infections in intensive care units reported that 7% cases were related to *Klebsiella pneumoniae* (Kolpa et al., 2018). *K. pneumoniae* is a common pathogen associated with indwelling medical device infections, especially catheter-associated urinary tract infections, catheter-related blood stream infections, and ventilator-associated pneumonia (Singhai et al., 2012; Percival et al., 2015). *K. pneumoniae* can form biofilms on abiotic and human tissue surfaces (Reid et al., 1992; Clegg and Murphy, 2016). The capsular polysaccharide (CPS) and fimbriae of *K. pneumoniae* are reported to be the important factors contributing to biofilm formation (Vuotto et al., 2014; Chung, 2016). However, in different studies, results regarding the role of the CPS in biofilm formation by *K. pneumoniae* are conflicting (Vuotto et al., 2014; Wang et al., 2017). Genes encoding type 1 and type 3 fimbriae are common and well characterized in most *K. pneumoniae* strains. Type 1 fimbriae are found in the majority of Enterobacteriaceae family members. The type 1 fimbriae of *K. pneumoniae* are encoded by the genes in the *fimAICDFGHK* operon. The FimA protein constitutes the major fimbrial subunit. FimH is a mannose-binding adhesin (Struve et al., 2008). Type 3 fimbriae are produced by many members of the Enterobacteriaceae (Sebghati et al., 1998; Ong et al., 2010). The components of type 3 fimbriae are encoded by the genes in the *mrkABCDF* operon (Allen et al., 1991). The gene *mrkA* encodes the major fimbrial subunit that is polymerized to form the fimbrial shaft. MrkD is an adhesin located at the fimbrial tip (Chung, 2016). Type 1 fimbriae are essential for *K. pneumoniae* to cause urinary tract infections (Rosen et al., 2008; Struve et al., 2008). Type 3 fimbriae are reported to mediate the attachment of *K. pneumoniae* to the extracellular matrix, bind to human endothelial and bladder cells and promote biofilm formation on biotic and abiotic surfaces (Tarkkanen et al., 1997; Jagnow and Clegg, 2003; Schroll et al., 2010).

Cyclic 3′5′-adenosine monophosphate (cAMP) is a second messenger found in all cellular organisms and involved in global gene regulation (Lin and Green, 1989). cAMP is catalyzed from ATP by a group of enzymes known as adenylyl cyclases (ACs). These enzymes are divided into six classes based on their primary structures. *Escherichia coli* possesses a single class I AC (Cya). *Mycobacterium tuberculosis* H37Rv possesses at least 16 class III AC-like proteins, while *Pseudomonas putida* possesses a CyaA-type AC. cAMP binds to and then activates the transcriptional factor cAMP receptor protein (CRP), also called catabolite gene activator protein (CAP) (Green et al., 2014). The CRP-cAMP homodimer binds to CRP binding sites (TGTGA-N6-TCACA or TGCGA-N6-TCGCA) to enhance the ability of the RNA polymerase holoenzyme to initiate gene transcription (Gunasekera et al., 1992). The genome of *E. coli* contains approximately 200 CRP regulons. In addition to regulating genes responsible for carbon metabolism, CRP-cAMP regulates various stress-related genes, such as chaperone proteins and cold shock and heat shock proteins (Gosset et al., 2004; Zheng et al., 2004). In *K. pneumoniae* MGH78578, 198 operons including 378 genes are predicted to be regulated by CRP (Novichkov et al., 2010). Lin et al. (2016) reported that CRP indirectly repressed *mrkA* transcription via repression of *mrkHI* transcription in *K. pneumoniae* CG43S3. In *K. pneumoniae* AJ218 and *K. pneumoniae* IApc35, *mrkH* which is in *mrkHI* bicistronic operon and located immediately adjacent to the *mrkABCDF* operon encodes a transcriptional activator that contains PilZ domain which mediates c-di-GMP binding and DNA binding. MrkH directly activates transcription of *mrkA* and its own expression by binding to the region close to the promoter in the presence of c-di-GMP (Wilksch et al., 2011; Tan et al., 2015; Schumacher and Zeng, 2016). MrkI, a LuxR-type transcriptional regulator, is a co-activator for the expression of *mrkA*. However, overexpression of *mrkH* in a *mrkI* mutant background could restore fimbrial expression (Johnson et al., 2011).

Bacteria regulate diverse aspects of physiology in response to the carbohydrate availability via the phosphoenolpyruvate (PEP):carbohydrate phosphotransferase system (PTS), which is a phosphorylation cascade that transfers phosphate sequentially from PEP to substrates (carbohydrates). Enzyme I (EI) and the histidine phosphocarrier protein (HPr) are the general cytoplasmic PTS proteins involved in the translocation of all PTS carbohydrates in most bacteria, whereas substrate specificity depends on the enzyme II (EII) complex. In most PTSs, the EII complex consists of membrane-bound EIIC component/domain and cytoplasmic EIIA and EIIB component/domain. Bacteria contain more than one EII complex; for example, *E. coli* contains at least 15 different EII complexes. In addition to regulating sugar (carbohydrates) transport, PTSs have been reported to regulate bacterial potassium uptake, nitrogen source utilization, and carbohydrate metabolic programs such as carbohydrate catabolite repression (CCR) and inducer exclusion (Deutscher et al., 2006, 2014). The glucose-specific EII complex of enteric bacteria consists of two distinct proteins: the cytoplasmic protein EIIA^Glc^, encoded by *crr*, and the membrane-associated protein EIICB^Glc^, encoded by *ptsG*, which contains the hydrophilic EIIB domain and the membrane-associated EIIC domain. EIIA^Glc^ plays an important role in carbon metabolism in enteric bacteria not only by interacting with non-PTS permeases (such as LacY) to inhibit their activities but also by regulating AC to use glucose as a priority carbon source. These phenomena are called inducer exclusion and CCR, respectively. The phosphorylated or dephosphorylated state of EIIA^Glc^ determines its regulatory role. In the absence of glucose and in the presence of PEP, EIIA^Glc^ is mainly in a phosphorylated state and is required for the activation of AC. Unphosphorylated EIIA^Glc^ can bind to and inhibit several non-PTS proteins, such as LacY (a lactose permease), MelB (a melibiose carrier protein), MalK (an ATP-hydrolyzing component of the maltose transport system), and GlpK (a glycerol kinase) (Deutscher et al., 2006).

We previously found the putative EII complex – KPN00353 (EIIA homolog), KPN00352 (EIIB homolog), and KPN00351 (EIIC homolog) – in the genome of *K. pneumoniae* MGH78578 (Jeng et al., 2017; Horng et al., 2018). We found the *etcA*, *etcB*, and *etcC* (*etc* for enzyme two complex), homologous to *KPN00353*, *KPN00352*, and *KPN00351*, respectively, in *K. pneumoniae* STU1 in this study. Overexpression of *etcABC* enhanced biofilm formation and type 3 fimbriae synthesis in *K. pneumoniae* STU1. We identified a putative CRP binding site located upstream of *mrkA*. In addition, we found that overexpression of *etcABC* compensated for the role of EIIA^Glc^ in lactose uptake by *K. pneumoniae*. Furthermore, intracellular cAMP levels and the CRP transcriptional levels were observed in bacteria overexpressing *etcABC*. In summary, we provided a model to show the regulation of type 3 fimbriae by *etcABC* via the CRP-cAMP signaling pathway in *K. pneumoniae*.

## Materials and Methods

### Bacterial Strains, Plasmids, and Growth Conditions

The bacterial strains and plasmids used in this study are listed in Table 1. Primers are listed in Supplementary Table S1. Unless otherwise stated, *K. pneumoniae* and *E. coli* were routinely cultured in Luria-Bertani (LB) medium (10 g/L tryptone, 5 g/L yeast extract, and 10 g/L NaCl) supplemented with appropriated antibiotics at the following concentrations: kanamycin (50 μg/mL), ampicillin (100 μg/mL), chloramphenicol (100 μg/mL), and gentamicin (20 μg/mL) on the rotatory shaker at 37°C and 200 rpm. For observation of *lac* operon activity, 2 μL of diluted overnight cultured bacterial solution was inoculated on MacConkey plate without/with 1 mM cAMP and LB agar plate containing 50 μg/mL of 5-bromo-4-chloro-3-indolyl-β-galactopyranoside (X-gal). Overnight cultured bacteria were diluted 1000-fold into the M9 minimal medium supplemented with 1% lactose and incubated for 24–48 h. The two clinical isolates of *K. pneumoniae* from bacterial storage bank in Tzu Chi Hospital to College of Medicine at Tzu Chi University was through official transfer.

**Table 1:** Bacterial strains and plasmids used in this study.

Strain	Relevant genotype and phenotype	Reference or source
*E. coli*		
DH5α	F^-^, φ80d*lacZ*ΔM15 (*lacZYA-argF*) U169, *deoR*, *recA1*, *endA1*, *hsdR17*(rk^-^, m_k_^+^), *phoA*, *supE44*, *λ*^-^, thi-1, *gyr*A96, *relA*1	Invitrogen
S17-1 λpir	λ-pir lysogen of S17-1 [*thi* *pro* *hsdR*^-^ *hsdM*^+^ *recA* RP4 2-Tc::Mu-Km::Tn7 (TpR. SmR.)]. Permissive host able to transfer suicide plasmids requiring the Pir protein by conjugation to recipient cells	Simon et al., 1983
BL21(DE3)pLysS	F^-^, *ompT*, *gal*, *dcm*, *lon*, *hsdS_B_*(r_B_^-^m_B_^-^), *λ*(DE3), pLysS(cm^R^)	Novagen
*K. pneumoniae*		
STU1	Laboratory-maintained strain, Amp^r^	National Taiwan University
Δ*crr*	In frame deletion of *crr* gene in STU1	This work
Δ*cyaA*	In frame deletion of *cyaA* gene in STU1	This work
Δ*crp*	In frame deletion of *crp* gene in STU1	This work
Δ*crr*Δ*etcABC*	In frame deletion of *etcABC* in Δ*crr*	
Clinical Kp-1	Clinical strain labeled Kp 083535 which was isolated from blood specimen	Tzu Chi Hospital, Hualien, Taiwan
Clinical Kp-2	Clinical strain labeled Kp 036749 which was isolated from urine specimen	Tzu Chi Hospital, Hualien, Taiwan
**Plasmid**		
pET30b	Vector, Km^r^	Novagen (Merck, Darmstadt, Germany)
pET30b::mrkA	pET30b derivative carrying structure gene of *mrkA* to express His-tagged MrkA, Km^r^	This work
pET30b::fimA	pET30b derivative carrying structure gene of *fimA* to express His-tagged FimA, Km^r^	This work
pET30b::crp	pET30b derivative carrying structure gene of *crp* to express His-tagged CRP, Km^r^	This work
pUT::minTn5-km1	Suicide plasmid requiring the Pir protein for replication and containing a mini-Tn5 cassette containing Km^r^ gene, Ap^r^, Km^r^	de Lorenzo et al., 1990
pBlueScript SK+ (pBSK)	Vector containing *lac* promoter, pUC *ori*, Amp^r^	Stratagene (CA, United States)
pBSK-Gm	pBSK derivative carrying gentamicin resistance gene at the *Sca*I site, Gm^r^	Horng et al., 2018
pBSK::Gm::etcABC	pBSK-Gm derivative carrying complete *etcABC* (promoter and structure), Gm^r^	This work
pBSK::Gm::cyaA	pBSK-Gm derivative carrying complete *cyaA*, Gm^r^	This work
pBSK::Gm::crr	pBSK-Gm derivative carrying complete *crr*, Gm^r^	This work
pBAD33	Vector utilizing P_BAD_ promoter, pACYC184 *ori*, Cm^r^	Guzman et al., 1995
pBAD33::crp	pBAD33 derivative carrying complete *crp*, Cm^r^	This work
pK18mobsacB	Vector, pBR322 *ori* (*ori* V), RP4 mob, *sacB*, Km^r^	Schafer et al., 1994
pW18mobsacB	pK18mobsacB derivative that R6K *ori* with *Bam*HI fragment was inserted into and pBR322 *ori* was removed from by using *Sac*I restriction enzyme; a suicide plasmid in *K. pneumoniae*, Km^r^	This work
pKO-Crp	yhfA’ (upstream of *crp* gene) and yhfK’ (downstream of *crp* gene) were inserted into pW18mobsacB with *Sma*I site, Km^r^	This work
pACYC184	Vector, p15A *ori*, Tc^r^, Cm^r^	Chang and Cohen, 1978
pACYC184-Sm^r^	pACYC184 derivative carrying streptomycin resistant gene in *Hin*dIII site, Sm^r^	This work
pPless-lux	pACYC184-Sm^r^ derivative carrying promoterless *luxCDABE* in *Bam*HI site, Sm^r^	This work
pPcrp-lux	pPless-lux derivative carrying *crp* promoter in front of *luxCDABE*, Sm^r^	This work


### Quantification of Biofilm Formation

The biofilm formation assay was conducted according to a previous published protocol with some modifications (O’Toole and Kolter, 1998). In brief, 2 mL of a bacteria suspension was inoculated into a Falcon polystyrene tube after dilution of the bacteria from an overnight culture with fresh LB to optical density at 600 nm (OD_600_) of 0.1. After incubation at 37°C for 24 h, the value of OD_600_ of the bacterial culture in the tube was measured. Thereafter, the bacterial suspension was discarded and the tube was rinsed twice with water. After incubation of 3 mL of 0.1% crystal violet at room temperature for 20 min, the tube was rinsed twice with water followed by air-drying. After addition of 95% ethanol, the absorbance of the ethanol solution was measured at 590 nm.

### Construction of Gene Deletion Mutants

For unmarked mutagenesis in *K. pneumoniae*, we constructed a suicide vector, pW18mobsacB, based on the *E. coli* plasmid, pK18mobsacB (Schafer et al., 1994). In brief, R6K*ori* cut from the plasmid, pUT::minTn5-km1 (de Lorenzo et al., 1990), was inserted into *Bam*HI site of pK18mobsacB and then pBR322 *ori* (*ori*V) was eliminated using *Sac*I restriction enzyme to form the suicide vector, pW18mobsacB, which remained *oriT*_RP4 for RP4-mediated conjugation, *sacB* for negative selection and *kan* for kanamycin resistance. For specific gene deletion in *K. pneumoniae*, the approximately 700-bp upstream and downstream flanking DNA fragments of the specific gene were, respectively, amplified. These two fragments were inserted into pW18mobsacB. The plasmid, pW18mobsacB containing flanking DNA fragments, was transferred from *E. coli* S17-1 λ pir to *K. pneumoniae* by conjugation (Soo et al., 2007). The transconjugant was spread on LB plates containing ampicillin (100 μg/mL) and kanamycin (50 μg/mL) for positive selection. Subsequently, the colonies from the positive selection were subcultured into LB broth containing 20% sucrose for negative selection. Then, the overnight bacterial culture was diluted and spread on LB plate containing 20% sucrose for getting the single colony of mutant candidate. The mutant strains were confirmed by PCR, followed by sequencing.

### Cell Culture and Adhesion Assay

Cell culture and adhesion assay were performed as described previously with some modification (March et al., 2011). Human lung carcinoma cells A549 (ATCC CCL185) were grown to 90% confluence in F-12K medium supplemented with 10% fetal bovine serum, 100 units/mL penicillin and 100 μg/mL streptomycin in 24-well cell culture dishes (4 × 10^4^ cells per well) at 37°C under a humidified 5% CO_2_ atmosphere. For the adhesion assays, A549 cells were washed three times with phosphate-buffered saline (PBS) and then infected with a suspension of 2 × 10^7^ bacterial cells in F12K medium. After 3-h infection, cells were washed three times with PBS. Subsequently, the adhered bacteria were released by 500 μL of 0.5% Triton X-100 for 5 min and serial dilutions were plated on LB agar plates for viable counts of bacteria.

### Western Blotting

The bacterial concentration was determined by measuring the optical density (OD) at 600 nm. A fixed amount of bacteria was collected by centrifugation, re-suspended in SDS sample buffer and then lysed by heating for 10–15 min at 100°C. An aliquot of total bacterial proteins was analyzed by 12% SDS polyacrylamide gel (SDS-PAGE) and transferred to a nitrocellulose membrane, Amersham Hybond-C Extra (GE Healthcare, IL, United States), by Amersham Mini Trans-Blot semiphor transphor unit (GE Healthcare, IL, United States). The detection procedures were performed as described in the previous study using Amersham ECL Prime Western Blotting Detection reagent (GE Healthcare, IL, United States) (Jeng et al., 2017). The intensities of the bands were detected using the gel catcher 2850 chemiluminescence camera system (CLUBIO, Taipei, Taiwan). For detection of MrkA, FimA, and mannose 6-phosphate isomerase (ManA), rabbit polyclonal antibody specific to the protein was the first antibody (LTK BioLaboratories, Taoyuan, Taiwan) and peroxidase-conjugated anti-rabbit IgG antibody was the second antibody (GE Healthcare, IL, United States). Rabbit anti-MrkA and anti-FimA polyclonal antibody were produced using His tagged proteins as immunogens in this study. Anti-ManA rabbit polyclonal antibody was produced in the previous study (Soo et al., 2014). For detection of CRP, mouse monoclonal antibody specific to CRP was the first antibody (BioLegend, CA, United States) and peroxidase-conjugated anti-mouse IgG antibody was the second antibody (Sigma-Aldrich, MO, United States).

### Electrophoretic Mobility Shift Assay (EMSA)

DNA fragments for EMSA were amplified by PCR and using the specific primers (Supplementary Table S1). Purified His-tagged CRP was dialyzed using the dialysis buffer (400 mM NaCl, 25% glycerol, 10 mM DTT, 20 mM Tris–HCl, pH 7.5). For the serial dilution experiments, CRP protein was serially diluted in binding reaction buffer (20 mM Tris–HCl [pH 8], 0.1 mM MgCl_2_, 150 mM KCl, and 0.05 mM EDTA, 12.5% glycerol). The binding reaction comprised His-tagged CRP protein and DNA fragments was performed in binding reaction buffer supplemented with 30 μg/mL poly(dI-dC) and 1 μg/μL bovine serum albumin. The reaction mixtures were incubated for 30 min at room temperature before being loaded onto 7% non-denaturing polyacrylamide gels containing 0.5 × Tris-borate-EDTA buffer. After electrophoresis at 100 V for 1 h, the gel was stained with ethidium bromide solution.

### Purification of His-Tagged Proteins

To purify His-tagged MrkA and His-tagged FimA for producing anti-MrkA and anti-FimA and to purify His-tagged CRP for EMSA, Novagen’s pET30b (Merck, Darmstadt, Germany) containing the structure gene of *mrkA*, *fimA*, or *crp*, respectively, was transformed to *E. coli* BL21(DE3) pLysS. After bacteria were cultured to mid-logarithmic phase, 0.5 mM isopropyl β-D-1-thiogalactopyranoside (IPTG) was added into the culture, followed by further incubation for 3–4 h at 30°C. Bacterial cells were collected by centrifugation and then suspended in the LEW solution containing 30 mM imidazole. The preparation of LEW solution and protein purification using Protino Ni-TED 1000 Packed Columns followed the manufacturer’s instructions (Macherey-Nagel, Düren, Germany).

### Quantitative Reverse Transcription PCR (RT-qPCR)

The bacterial RNA was extracted using TRI reagent (Sigma-Aldrich, MO, United States) and treated using RNase-free DNase I (New England Biolabs, MA, United States) for 30 min at 37°C to remove the DNA contamination. RNA was reversed transcribed by using QuantiTect reverse transcription kit (Qiagen, Hilden, Germany). Quantitation of cDNA from the transcripts of *fimA*, *fimH*, *mrkA*, *mrkD*, and *mrkH* was performed by real time PCR in triplicate by using the specific primers (Supplementary Table S1) and QuantiNova SYBR^®^ Green PCR Kit (Qiagen, Hilden, Germany) in the Rotor-Gene real-time genetic analyzer (Qiagen, Hilden, Germany). 16s rRNA was used as the internal reference gene for analysis.

### Transmission Electron Microscopy (TEM)

After 1 mL of overnight bacterial culture was centrifuged, the bacterial pellet was fixed in a primary fixation solution (2.5% glutaraldehyde, 0.1 M cacodylate buffer, and 1% tannic acid) at room temperature for 1 h. Thereafter, 10 μl of bacterial suspension was absorbed onto 200-μm-pore-size mesh copper electron microscopy grids coated with carbon and Formvar. Subsequently, the grid was floated on a drop of 2% (w/v) uranyl acid for 15 s to negatively stain the bacterial sample. Bacterial cells were observed under a Hitachi H-7500 transmission electron microscope (Hitachi, Japan) operated under standard conditions with the cold trap in place. For immunogold electron microscopy, the bacteria were fixed with primary fixation solution and washed with PBS. After washed, the bacteria were blocked in blocking solution [5% w/v of bovine serum albumin (BSA) in PBS and 1% Tween 20] for 30 min. Subsequently, bacteria were incubated with rabbit anti-MrkA polyclonal antibody diluted in blocking solution (1:50) for 30 min at room temperature. After being washed three times with blocking solution, the bacteria were incubated with (goat) colloidal gold particle-conjugated anti-rabbit IgG (Sigma-Aldrich in Merck, Germany) for 30 min at room temperature. Before post-fixation (2% glutaraldehyde in PBS), the bacteria were washed twice with PBS. Ten microliters of bacterial suspension was absorbed onto carbon-coated grids for 2–3 min under a light bulb. After excess liquid was removed, the grids were rinsed with distilled water and negatively stained with 2% phosphotungstic acid (PTA) for 1 min. Samples were viewed on TEM.

### Quantification of cAMP

After overnight culture, the bacterial OD was measured at 600 nm. A fixed amount of bacteria suspended in the lysis buffer (0.1 M HCl and 0.5% Triton X-100) was stored overnight at -70°C. After sonication and centrifugation, the amount of cAMP in the supernatant was measured by using the non-acetylated format provided in Direct cAMP ELISA kit (Enzo Life Sciences, NY, United States). All procedures followed the protocols supplied by the manufacturer.

### Quantification of CRP Promoter Activity

To construct the *luxCDABE* reporter plasmid, the *luxCDABE* was cut from pBG (Soo et al., 2008) and subsequently inserted into pACYC184-Sm^r^ to form pPless-lux (Table 1). The 400-bp fragment containing *crp* promoter was amplified by PCR using primers pair, crp promoter FP/crp promoter RP (Supplementary Table S1), then digested with *Bam*HI/*Eco*RV and inserted in front of *luxCDABE* in pPless-lux, forming pPcrp-lux (Table 1). Either pPless-lux or pPcrp-lux was transferred into Δ*crr*Δ*etcABC*, *K. pneumoniae* with double mutation of *crr* and *etcABC*, by electroporation. To observe the effect of EtcABC overexpression on CRP promoter activity, either pBSK::Gm::etcABC or pBSK-Gm was transferred into the strains, Δ*crr*Δ*etcABC* carrying pPless-lux or pPcrp-lux. After overnight culture in LB containing 0.5 mM IPTG, the bioluminescence was measured for 10 s by using Modulus single tube multimode luminescence value reader (Turner BioSystems, CA, United States). All procedures followed the protocols supplied by the manufacturer.

### Nucleotide Sequence Accession Number

The nucleotide sequences of the *etcABC* genes have been deposited at GenBank nucleotide sequence database under the accession numbers MK675058.

### Statistical Methods

Results of RT-qPCR, the concentration of intracellular cAMP and *crp* promoter activity were expressed as mean ± standard deviation from three independent experiments. Paired Student’s *t*-test was performed to determine statistically significant differences, and *p*-values of <0.05 were considered to indicate statistical significance.

## Results

### Overexpression of *etcABC* Enhanced Type 3 Fimbriae Production and Biofilm Formation

**FIGURE 1:**
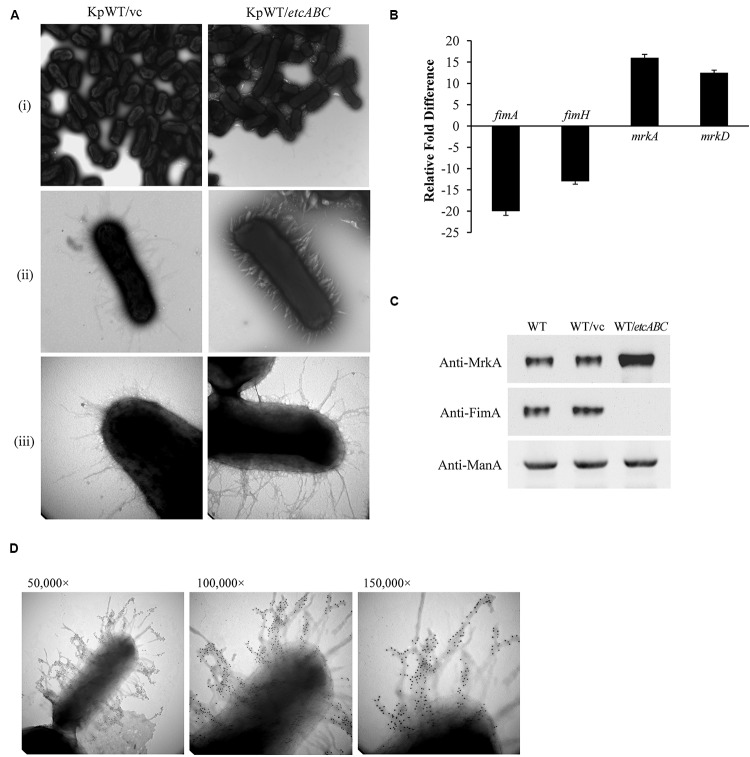
Overexpression of *etcABC* enhanced type 3 fimbriae production in *K. pneumoniae*. **(A)** The representative TEM images of bacteria incubated in LB broth in three independent experiments. KpWT/vc: *K. pneumoniae* STU1 carrying pBSK-Gm as the vector control. KpWT/*etcABC*: *K. pneumoniae* STU1 carrying pBSK::Gm::*etcABC* to overexpress *etcABC*. The magnifications of the images are **(i)** 15,000×, **(ii)** 50,000×, and **(iii)** 100,000×. **(B)** Transcriptional analysis of *fimA*, *fimH*, *mrkA*, and *mrkD* in KpWT/*etcABC* and KpWT/vc by RT-qPCR. The 16S rRNA gene was used as the reference. Relative gene expressions means amount of *mrkA* and *mrkD* mRNA in KpWT/*etcABC* compared to those in KpWT/vc and amount of *fimA* and *fimH* mRNA in KpWT/vc compared to those in KpWT/*etcABC*. The presented results are the means ± standard deviations of three replicates. The *p*-value of each group is less than 0.05 as compared with KpWT/vc. **(C)** Western blot analysis of MrkA, FimA, and ManA expression in each strain. WT: *K. pneumoniae* STU1, WT/vc: *K. pneumoniae* STU1 carrying pBSK-Gm as the vector control. WT/*etcABC*: *K. pneumoniae* STU1 carrying pBSK::Gm::etcABC to overexpress *etcABC*. ManA was used as the loading control. The blots are representatives of at least three independent experiments. **(D)** Immunogold electron microscopy using anti-MrkA was performed against *K. pneumoniae* STU1 carrying pBSK::Gm::etcABC. Bacteria displayed type 3 fimbriae. The magnification of the images is 50,000× (left), 100,000× (middle), and 150,000× (right), respectively.

Our previous data showed that overexpression of KPN00353-KPN00352-KPN00351 increased biofilm formation by *K. pneumoniae* MGH78578 (Horng et al., 2018). In STU1, we found homologs having high sequence identity to *KPN00353* (98%), *KPN00352* (100%), and *KPN00351* (99%) of MGH78578, respectively. We therefore refer to these genes as *etcA*, *etcB*, and *etcC* in this manuscript and the effect of EtcABC on biofilm formation was observed. Overexpression of *etcABC* in *K. pneumoniae* STU1 and two clinical *K. pneumoniae* isolates resulted in an increase in biofilm formation (Supplementary Figure S1A). The two clinical strains were isolated from blood and urine specimens, respectively (Table 1). Like STU1, these two clinical strains also have KPN00353-KPN00352-KPN00351 homologs, examined by PCR (data not shown). In addition, the effects of EtcABC overexpression on adhesion of *K. pneumoniae* STU1 to A549 epithelial alveolar cells were also examined. The amount of *K. pneumoniae* STU1 overexpressing *etcABC* adhered on A549 cells was more than that of *K. pneumoniae* STU1 carrying vector control (Supplementary Figure S1B). Fimbriae are reported to be an important factor contributing to bacterial biofilm formation and adhesion to cells (Hornick et al., 1992; Tarkkanen et al., 1997; Chung, 2016). Therefore, in this study, we examined the effects of *etcABC* on *K. pneumoniae* fimbriae production. Transmission electron microscopy (TEM) observation of bacteria grown in LB broth showed a greater presence of peritrichous pili on the surface of *K. pneumoniae* overexpressing *etcABC* than on the surface of the vector control (Figure 1A). However, the mutant strain lacking *etcABC* did not exhibit a difference from the wild-type strain (data not shown). To verify the type of pili expressed in *K. pneumoniae* overexpressing *etcABC*, we sought to quantify the transcriptional and translational levels of type 1 and type 3 fimbriae. Quantitative reverse transcription PCR (RT-qPCR) analysis showed that the mRNA levels of *fimA* and *fimH* were markedly decreased in *K. pneumoniae* overexpressing *etcABC* relative to their levels in the vector control. By contrast, the transcriptional levels of *mrkA* and *mrkD* in *K. pneumoniae* overexpressing *etcABC* were higher than those in the vector control (Figure 1B). Immunoblots of whole cell extracts of *K. pneumoniae* overexpressing *etcABC* probed with anti-MrkA polyclonal antibodies showed that MrkA, the component of the type 3 fimbrial shaft, was highly expressed relative to its expression in the vector control. However, *K. pneumoniae* overexpressing *etcABC* did not express FimA (Figure 1C). The amount of MrkA expressed by the mutant strain with the *etcABC* deletion was not different from that of the wild-type strain (data not shown). The expression of mannose 6-phosphate isomerase (ManA) in each strain confirmed the consistent sampling of bacteria (Soo et al., 2014). The fimbriae on the surface of *K. pneumoniae* overexpressing *etcABC* appeared to be in bundle or thicker than vector control (Figure 1A). To corroborate that fimbriae of *K. pneumoniae* overexpressing *etcABC* were type 3 fimbriae, the immunogold electron microscopy was performed. The immunogold electron microscopy micrograph of *K. pneumoniae* overexpressing *etcABC* using anti-MrkA showed gold particle localization on the fimbriae (Figure 1D). These results indicated that overexpression of *etcABC* positively regulated the *mrk* operon to enhance type 3 fimbriae production in *K. pneumoniae*.

### CRP Positively Regulated Type 3 Fimbriae Production

**FIGURE 2:**
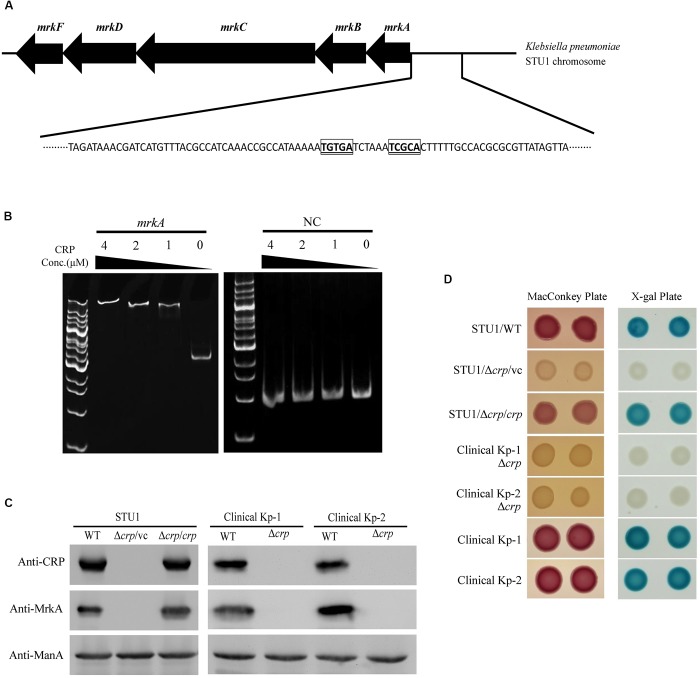
The regulation of MrkA expression by CRP in *K. pneumoniae*. **(A)** The genetic organization of the *mrk* operon and putative CRP binding site located upstream of *mrkA*. The putative CRP core binding sites are indicated with bolded, underlined and boxed letters. **(B)** The EMSA demonstrating the interaction of the region upstream of *mrkA* and CRP. *mrkA*: a 394-bp DNA fragment containing the *mrkA* upstream region. NC: a 222-bp DNA fragment containing the EtcA structural region as the negative control. The concentration of purified His-tagged CRP ranged from 4 to 1 μM. The gel is representative of three independent experiments. **(C)** Western blot analysis of CRP, MrkA, and ManA expression in *K. pneumoniae*. ManA was used as the loading control. The blots are representatives of at least three independent experiments. **(D)** The representative colony phenotypes on MacConkey agar (MacConkey plate) and LB agar containing X-gal (X-gal plate) in three independent experiments. WT, wild-type *K. pneumoniae*. Δ*crp*: *crp* mutant. Δ*crp*/vc: *crp* mutant carrying pBAD33 as the vector control. Δ*crp/crp*: complementation strain of the *crp* mutant carrying pBAD33::crp. Clinical Kp-1 and Clinical Kp-2 are two clinical *K. pneumoniae* isolates. The phenotypes of the *K. pneumoniae* STU1 *crp* mutant (Δ*crp*) and its corresponding vector control (Δ*crp*/vc) were the same, so Δ*crp*/vc was used to represent of *K. pneumoniae* STU1 Δ*crp* in this figure.

To identify possible factors regulating the transcription of the *mrk* operon, we performed sequence analysis and found a putative CRP binding site in the region upstream of the *mrk* operon (Figure 2A). An electrophoretic mobility shift assay (EMSA) was performed to confirm the binding of CRP to the region upstream of *mrkA* (Figure 2B). To confirm whether CRP regulated the expression of the *mrk* operon, the *crp* gene was deleted in *K. pneumoniae* STU1 and two clinical *K. pneumoniae* isolates to assess MrkA expression in *crp* mutant strains (Supplementary Figure S2). All three *crp* mutants and one complementation strain of the *K. pneumoniae* STU1 *crp* mutant strain were confirmed by Western blotting using anti-CRP monoclonal antibodies. None of the three *crp* mutants expressed MrkA, but all three of the wild-type and one complementation strain did (Figure 2C). In addition, the amount of *mrkA* transcript was reduced in all of three *crp* mutants containing vector, compared to their parent stains containing vector. The amount of *mrkA* transcript was restored in all of three *crp* complementation strains (Supplementary Figures S3A–C). These results indicated that CRP positively regulated type 3 fimbrial shaft synthesis in *K. pneumoniae*. Since fimbriae are important for bacterial biofilm formation and adhesion to cell, the effect of CRP on *Klebsiella* biofilm formation and adhesion to cell was observed. Even though the growth rate was slightly reduced, the *K. pneumoniae* STU1 *crp* mutant and *crp* mutant carrying vector showed the dramatical defects in both biofilm formation and adhesion to A549 cells, compared to wild type, *K. pneumoniae* STU1. The biofilm formation and adhesion ability were restored in *crp* complementation strain (Supplementary Figure S4). Therefore, these results suggested that CRP positively regulated type 3 fimbriae production in *K. pneumoniae*.

CRP-cAMP was reported to be an activator of lactose fermentation in *K. pneumoniae* (formerly *K. aerogenes*) (Baldauf et al., 1988). To confirm the role of CRP in regulating the *lac* operon of the three *K. pneumoniae* strains in this study, the three *crp* mutants, one *K. pneumoniae* STU1 *crp* mutant carrying the vector (pBAD33; Δ*crp*/vc), three wild-type strains (*K. pneumoniae* STU1 and two clinical strains) and the complementation strain (*K. pneumoniae* STU1 Δ*crp* carrying pBAD33::crp; Δ*crp*/*crp*) were grown on MacConkey agar and LB agar containing X-gal. The three wild-type strains and the complementation strain formed pink colonies on MacConkey agar and blue colonies on LB agar containing X-gal. In contrast, the *crp* mutants formed colorless colonies on MacConkey agar and white colonies on LB agar containing X-gal (Figure 2D). These results indicated that CRP positively regulated the *lac* operon in these three *K. pneumoniae* strains.

### Regulation of MrkA by *etcABC* Was Dependent on CRP

**FIGURE 3:**
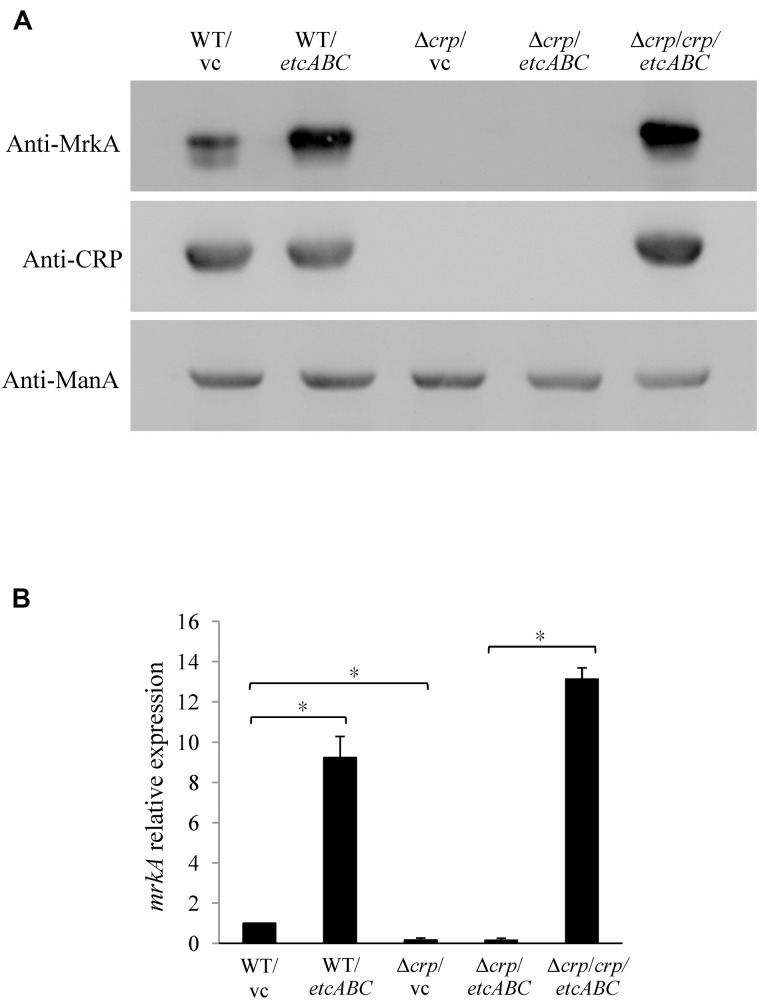
The effects of EtcABC overexpression on MrkA was dependent on CRP. **(A)** Western blot analysis of MrkA, CRP, and ManA expression in each strain grown in LB. ManA was used as the loading control. The blots are representatives of at least three independent experiments. **(B)** RT-qPCR analysis of *mrkA* transcription in each strain grown in LB. The 16S rRNA gene was used as the reference. The transcriptional level of *mrkA* in each strain was compare to WT/vc. The presented results are the means ± standard deviations of three replicates. An asterisk (^∗^) represents *p* < 0.05 as compared with WT/vc or Δ*crp/crp/etcABC* compared with Δ*crp/etcABC*. WT/vc: *K. pneumoniae* STU1 carrying pBSK-Gm as the vector control. WT/*etcABC*: *K. pneumoniae* STU1 carrying pBSK::Gm::etcABC to overexpress *etcABC*. Δ*crp*/vc: *K. pneumoniae* STU1 *crp* mutant carrying pBSK-Gm as the vector control. Δ*crp/etcABC*: *K. pneumoniae* STU1 *crp* mutant carrying pBSK::Gm::etcABC to overexpress *etcABC*. Δ*crp/crp/etcABC*: *K. pneumoniae* STU1 *crp* mutant carrying pBAD33::crp and pBSK::Gm::etcABC. The phenotype of the *crp* mutant carrying the two plasmids pBAD33 and pBSK::Gm::etcABC was the same as that of the *crp* mutant carrying one plasmid, pBSK::Gm::etcABC.

To investigate the mechanism by which overexpression of *etcABC* enhanced type 3 fimbriae expression (Figure 1B,C), we examined the effects of *etcABC* on MrkA expression in the *crp* mutant. The Western blotting results showed that overexpression of *etcABC* in the wild-type strain, *K. pneumoniae* STU1, increased MrkA expression compared to that in the wild-type strain carrying the vector (Figure 3A). The RT-qPCR results showed that the amount of *mrkA* transcript was increased in *K. pneumoniae* STU1 overexpressing *etcABC*, compared to the vector control (Figure 3B). However, MrkA was not expressed in either the *crp* mutant containing the vector or the *crp* mutant overexpressing *etcABC* (Figure 3A). The *mrkA* mRNA level also reduced in *crp* mutant containing the vector and the *crp* mutant overexpressing *etcABC*, compared to *K. pneumoniae* STU1 carrying the vector (Figure 3B). The phenotype of the *crp* mutant carrying the two plasmids pBAD33 and pBSK::Gm::etcABC was the same as that of the *crp* mutant carrying one plasmid, pBSK::Gm::etcABC (data not shown). Overexpression of *etcABC* in the *crp* complementation strain, the *crp* mutant carrying the two plasmids pBAD33::crp and pBSK::Gm::etcABC, restored both *mrkA* transcript and MrkA production (Figure 3). MrkA expression in the mutant strain lacking *etcABC* was not different from that of the wild-type strain (data not shown). Since MrkH was reported to positively regulate the *mrkA* transcription, the effect of *etcABC* and *crp* on *mrkH* transcription was examined by RT-qPCR. The results showed that overexpression of *etcABC* increased *mrkH* transcription in wild type, but not in *crp* mutant. Besides, the mRNA of *mrkH* was reduced in *crp* mutant containing vector, compared to wild type containing vector. Overexpression of *etcABC* in the *crp* complementation strain restored *mrkH* transcript (Supplementary Figure S5). These results suggested that overexpression of *etcABC* regulated type 3 fimbriae via CRP.

**FIGURE 4:**
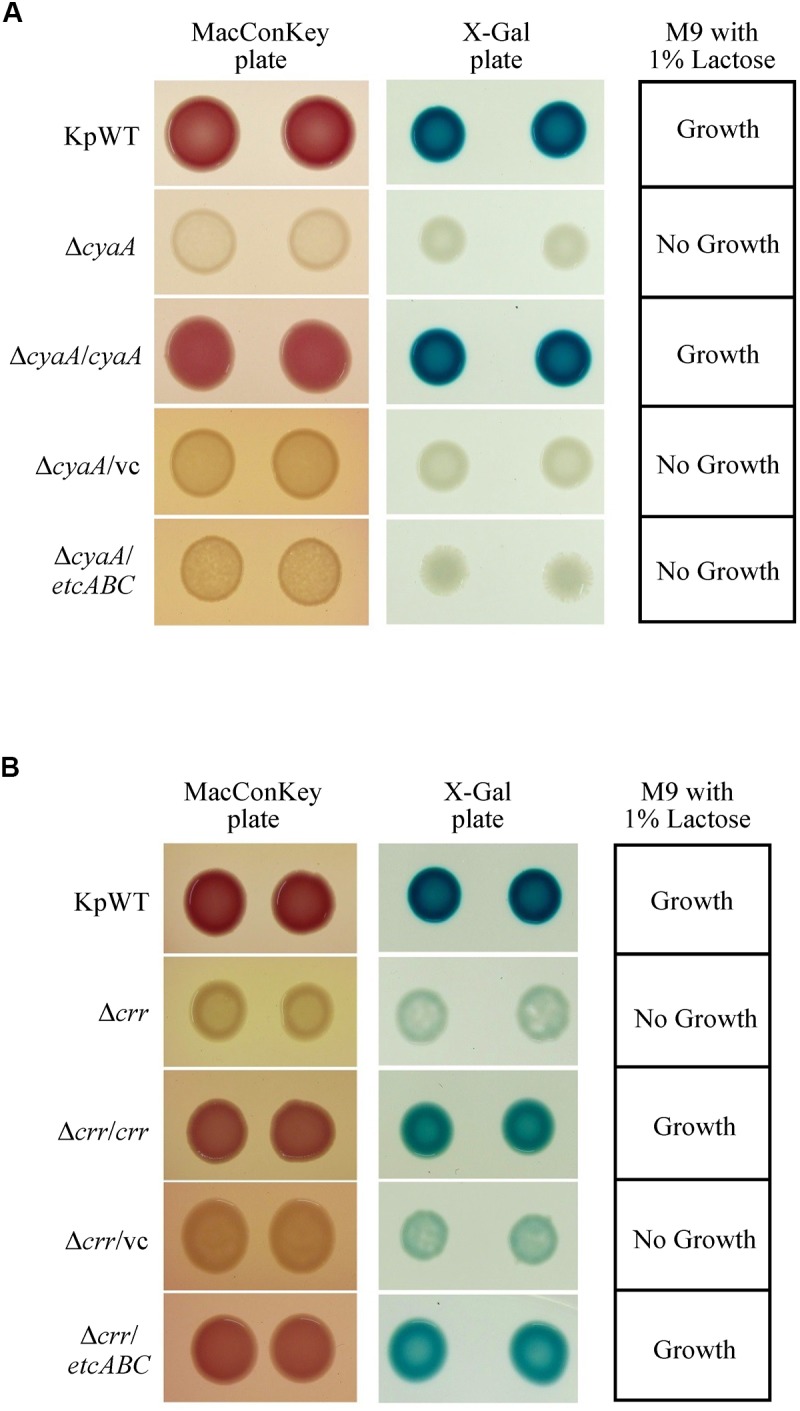
Colony morphology of *K. pneumoniae* STU1 and its derivative strains on MacConkey agar (MacConkey plate) and LB agar containing X-gal (X-gal plate) and bacterial growth in M9 minimal medium containing 1% lactose as the sole carbon source (M9 with 1% lactose) after overnight culture. KpWT: wild-type. **(A)** The phenotype of *cyaA* mutant and its derivative strains. Δ*cyaA*: *cyaA* mutant. Δ*cyaA/cyaA*: complementation strain of the *cyaA* mutant carrying pBSK::Gm::cyaA. Δ*cyaA*/vc: *cyaA* mutant carrying pBSK-Gm as the vector control. Δ*cyaA/etcABC*: *cyaA* mutant carrying pBSK::Gm::etcABC to overexpress *etcABC*. **(B)** The phenotype of *crr* mutant and its derivative strains. Δ*crr*: *crr* mutant. Δ*crr/crr*: complementation strain of the *crr* mutant carrying pBSK::Gm::crr. Δ*crr/*vc: *crr* mutant carrying pBSK::Gm as the vector control. Δ*crr/etcABC*: *crr* mutant carrying pBSK::Gm::etcABC to overexpress *etcABC*. The photos are representatives of three independent experiments.

**FIGURE 5:**
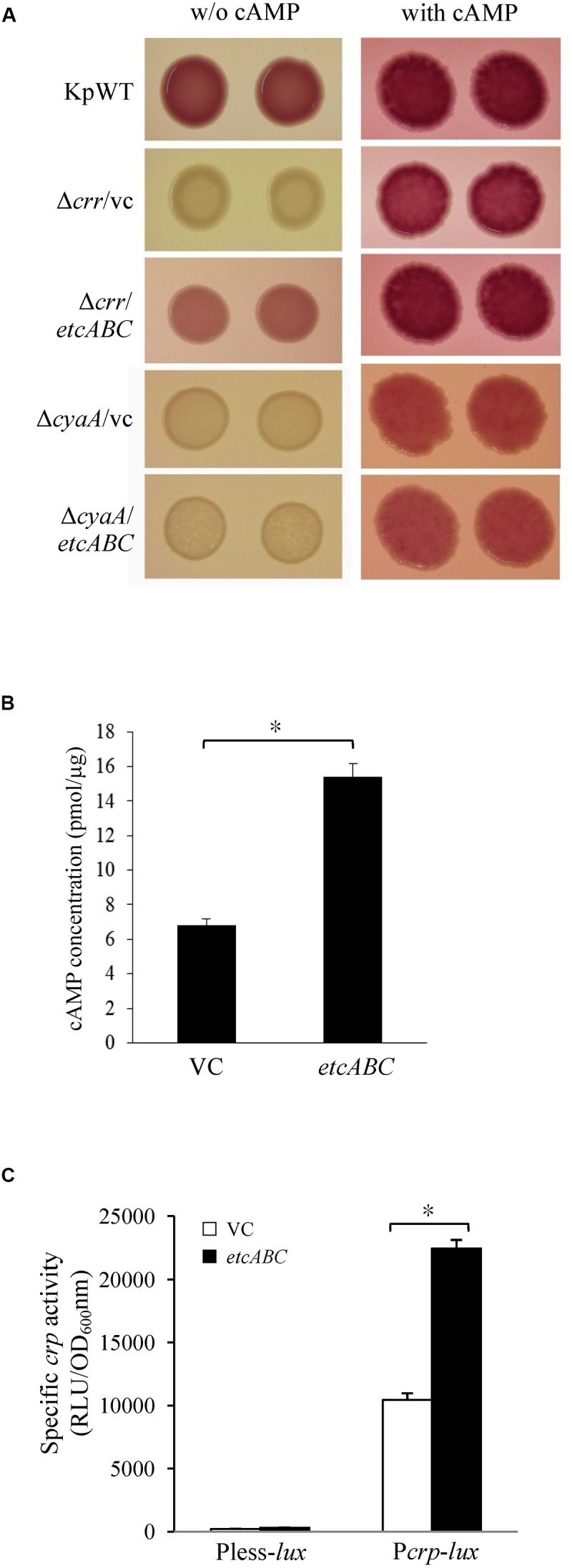
The effects of *etcABC* on cAMP production. **(A)** The representative colony morphology of *K. pneumoniae* STU1 and its derivative strains on MacConkey agar without (w/o) or with 1 mM cAMP in three independent experiments. KpWT: wild-type. Δ*crr*/vc: *crr* mutant carrying pBSK-Gm as the vector control. Δ*crr/etcABC*: *crr* mutant carrying pBSK::Gm::etcABC to overexpress *etcABC*. Δ*cyaA*/vc: *cyaA* mutant carrying pBSK-Gm as the vector control. Δ*cyaA/etcABC*: *cyaA* mutant carrying pBSK::Gm::etcABC to overexpress *etcABC*. **(B)** Quantification of intracellular cAMP levels by ELISA. **(C)** Quantification of *luxCDABE* luciferase activity in derivative strains of *K. pneumoniae* STU1. Pless-*lux*: Bacteria containing pPless-lux, a plasmid carrying a promoterless *luxCDABE*. P*crp*-*lux*: Bacteria containing pPcrp-lux, a plasmid carrying the *crp* promoter followed by the promoterless *luxCDABE*. VC in panels **(B,C)**: *K. pneumoniae* Δ*crr*Δ*etcABC* carrying pBSK-Gm as the vector control. *etcABC* in panels **(B,C)**: *K. pneumoniae* Δ*crr*Δ*etcABC* carrying pBSK::Gm::etcABC to overexpress *etcABC*. The data in panels **(B,C)** are presented as the averages ± standard deviations of at least three replicates. An asterisk (^∗^) represents *p* < 0.05 as compared with VC.

Binding of cAMP to CRP has been reported to increase the affinity and specificity of CRP for the target DNA in *E. coli* (Takahashi et al., 1980, 1989; Harman, 2001). EIIA^Glc^ can regulate the activity of adenylyl cyclase, also known as adenylate cyclase (AC or CyaA), to influence the cAMP concentration (Deutscher et al., 2006). Furthermore, EtcA was predicted to be an EIIA component. Therefore, we hypothesized that overexpression of *etcABC* can increase cAMP to enhance CRP activity in *K. pneumoniae*. Before testing this hypothesis, we examined whether the effects of cAMP on the *lac* operon in *K. pneumoniae* were the same as those in *E. coli* (Narang, 2009). The gene encoding CyaA was deleted, and growth of the *cyaA* mutant on MacConkey agar, on LB agar containing X-gal and in minimal medium containing lactose as the sole carbon source was observed. The *cyaA* mutant and its vector control formed colorless colonies on MacConkey agar and white colonies on LB agar containing X-gal and failed to grow in minimal medium containing lactose as the sole carbon source. In contrast, the wild-type and complementation strains formed pink colonies on MacConkey agar and blue colonies on LB agar containing X-gal and exhibited growth in minimal medium containing lactose as the sole carbon source (Figure 4A). Moreover, the phenotype of the *cyaA* mutant carrying the empty vector was restored by the addition of 1 mM cAMP to the MacConkey agar (Figure 5A). These results indicated that the effects of *cyaA* and cAMP on the *lac* operon were the same in both *K. pneumoniae* and *E. coli*.

### Overexpression of *etcABC* Compensated for the Role of *crr* in *lac* Operon Activation by CRP-cAMP Signaling

A *Salmonella typhimurium* mutant strain lacking *crr*, which encodes EIIA^Glc^, was reported to exhibit low levels of cAMP in medium containing galactose as the sole carbon source (Feucht and Saier, 1980). In this study, the *K. pneumoniae* *crr* mutant and its vector control formed very pale pink colonies on MacConkey agar and slightly blue colonies on LB agar containing X-gal and failed to grow in minimal medium containing lactose as the sole carbon source (Figure 4B). We assumed that deletion of *crr* resulted in a low level of cAMP in *K. pneumoniae*, leading to inactivation of the *lac* operon and, subsequently, pale colonies on MacConkey agar and LB agar containing X-gal, as well as death in medium containing lactose as the sole carbon source. To test this hypothesis, cAMP was added to MacConkey agar. The wild-type phenotype was restored in the *crr* mutant carrying the empty vector by the addition of cAMP (Figure 5A), indicating that Crr positively regulated the intracellular cAMP level in *K. pneumoniae*. However, overexpression of *etcABC* in the *crr* mutant restored the wild-type phenotype on MacConkey agar, LB agar containing X-gal and in minimal medium containing lactose as the sole carbon source but did not restore the wild-type phenotype in the *cyaA* mutant (Figure 4). This result strongly suggested that overexpression of *etcABC* compensated for the role of *crr* in CRP-cAMP-mediated activation of the *lac* operon.

### Overexpression of *etcABC* Increased Intracellular cAMP Levels

Overexpression of *etcABC* in the *cyaA* mutant did not result in pink colonies on MacConkey agar (Figure 4A, 5A). However, the addition of cAMP restored the wild-type phenotype in not only the *cyaA* mutant strain but also the *cyaA* mutant strain overexpressing *etcABC* (Figure 5A). This result indicated that cAMP production was dependent on AC. In a previous study, the phosphorylated form of Crr, EIIA^Glc^, was reported to stimulate the activity of AC (CyaA) in *E. coli* by protein–protein interactions, leading to increased cAMP production in medium lacking glucose (Deutscher et al., 2006). Moreover, EtcA is predicted to be an EIIA component. Therefore, we hypothesized that overexpression of *etcABC* can increase the activity of CyaA to elevate the level of cAMP. To test this hypothesis, we quantified the intracellular cAMP levels by ELISA and showed that compared to the vector control, *etcABC* overexpression in *K. pneumoniae* Δ*crr*Δ*etcABC* could increase intracellular cAMP levels (Figure 5B). We also examined the effects of *etcABC* overexpression on the intracellular cAMP levels in *K. pneumoniae* STU1 wild type and Δ*crr*. The results showed that *etcABC* overexpression in wild type and *crr* mutant also increased intracellular cAMP levels, compared to their corresponding vector control. However, the difference of cAMP levels between *etcABC* overexpression and vector control in Δ*crr*Δ*etcABC* was larger than those in wild type and *crr* mutant (Supplementary Figure S6).

cAMP receptor protein was reported to be positively autoregulated at the transcriptional level by CRP-cAMP in *E. coli* (Ishizuka et al., 1994). Therefore, a *crp* promoter-controlled *luxCDABE* reporter plasmid was constructed and transformed into *K. pneumoniae* Δ*crr*Δ*etcABC* to test the effect of exogenous cAMP on *crp* expression. The results showed that 10 min after cAMP was added, *crp* promoter activity increased relative to that in medium with no exogenous cAMP (Supplementary Figure S7), indicating that the transcriptional level of *crp* was dependent on cAMP in *K. pneumoniae*, as in *E. coli*. Subsequently, we tested the effect of *etcABC* overexpression on *crp* expression. Analysis of the bioluminescence emission revealed that the activity of the *crp* promoter in the strain overexpressing *etcABC* was twofold higher than that in the strain containing the vector (Figure 5C), indicating that overexpression of *etcABC* increased the transcriptional level of the *crp* gene. Thus, these results allowed us to conclude that overexpression of *etcABC* increased the levels of cAMP and activity of CRP-cAMP.

## Discussion

The knowledge about *lac* operon regulation by CRP-cAMP is mainly derived from studies in *E. coli* and *S. typhimurium*. The results of our study showed the same regulatory mechanisms in *K. pneumoniae*: (1) CRP and *cyaA* are essential for *lac* operon activation and lactose uptake (Figure 2D, 4A), and (2) *cyaA* is responsible for cAMP production (Figure 5). Furthermore, we observed that CRP positively regulated MrkA expression in *K. pneumoniae* STU1 grown in LB broth (Figure 2C). However, a conflicting result reported by Lin et al. (2016) showed that MrkA production in LB broth was markedly increased by deletion of *cyaA* or *crp* from the parent strain, *K. pneumoniae* CG43S3 and also reported that the mRNA levels of *mrkABCDF* genes and the activity of the *mrkA* promoter were increased in the *crp* mutant strain grown in LB broth. The authors suggested that CRP repressed type 3 fimbriae expression (Lin et al., 2016). After screening published reports, we found a report from Luo et al. (2017) showing that the transcriptional activity of the *KP1-4563* gene was increased in the *crp* mutant compared to that in the parent strain, *K. pneumoniae* NTUH-K2044. In addition, Luo et al. (2017) observed that the *KP1-4563* gene negatively regulated the function of type 3 fimbriae by indirect observation methods such as a mannose-resistant hemagglutination assay (MRHA) and bacterial adhesion assay. Although Luo et al. (2017) did not directly examine type 3 fimbriae synthesis in the *crp* mutant, they proposed that CRP positively regulated the function of type 3 fimbriae in *K. pneumoniae* NTUH-K2044. In addition, another research team found via yeast agglutination that CRP enhances fimbrial activity in *K. pneumoniae* NTUH-K2044 (Ou et al., 2017). To clarify the different findings about CRP and type 3 fimbriae, we randomly selected two clinical *K. pneumoniae* isolates for *crp* gene deletion. Neither of these two *crp* mutants grown in LB broth expressed MrkA, but their parent strains did (Figure 2C), suggesting that CRP positively regulated type 3 fimbriae synthesis in these two clinical *K. pneumoniae* isolates. Therefore, we believe that negative regulation of type 3 fimbriae by CRP is uncommon in *K. pneumoniae*.

**FIGURE 6:**
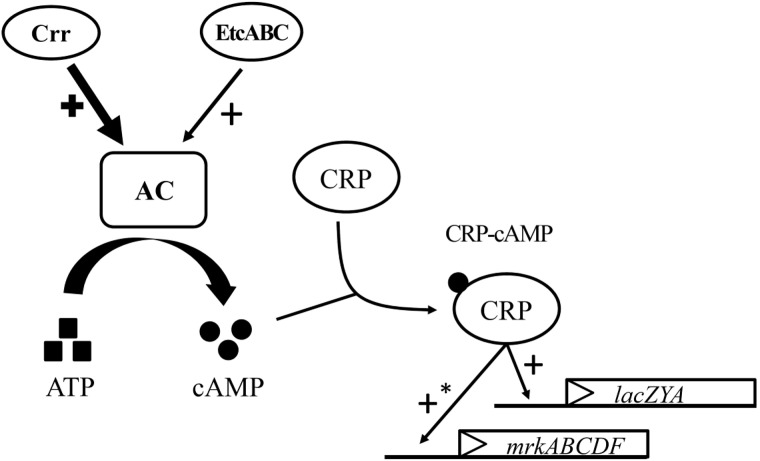
Model for the role of *etcABC* in the regulation of type 3 fimbriae synthesis and lactose uptake in *K. pneumoniae*. In LB medium, Crr and/or EtcABC activate AC to produce cAMP. Then, cAMP binds to CRP to enhance the transcriptional activity of CRP. CRP-cAMP increases the transcription of *lacZYA*, resulting in bacterial lactose transportation and metabolism. CRP-cAMP increases the transcription of *mrkABCDF*, resulting in an increase in type 3 fimbriae production. The effect of Crr on AC is much greater than that of EtcABC in LB medium. An asterisk (^∗^) means indirect regulation. The possible mechanism was discussed in the Section “Discussion.”

The *K. pneumoniae* *crr* mutant formed very pale pink colonies on MacConkey agar and slightly blue colonies on LB agar containing X-gal and failed to grow in minimal medium containing lactose as the sole carbon source (Figure 4B). The mutant strain lacking *etcABC*, like the wild-type strain, formed pink colonies on MacConkey agar and blue colonies on LB agar containing X-gal and exhibited growth in medium containing lactose as the sole carbon source (data not shown). The phenotype of the strain with the double mutation of *etcABC* and *crr* was similar to that of the *crr* mutant on MacConkey agar, on LB agar containing X-gal and in medium containing lactose as the sole carbon source (data not shown), indicating that compared to *etcABC*, *crr* played a more prominent role in the regulation of the *lac* operon by CRP-cAMP. However, overexpression of *etcABC* compensated for the role of *crr* in both the *lac* operon regulation phenotype and lactose uptake, which are mediated by CRP-cAMP. Moreover, overexpression of *etcABC* increased type 3 fimbriae synthesis, compared to the vector control (Figure 1, 3). However, type 3 fimbriae synthesis did not differ between the mutant strain lacking *etcABC* and the wild-type strain (data not shown). Therefore, we propose the following mechanism by which overexpression of *etcABC* enhances type 3 fimbriae synthesis in *K. pneumoniae* (Figure 6): in LB medium, the phosphorylation level of Crr, also known as EIIA^Glc^, is presumably high. Phosphorylated Crr enhances the activity of AC to produce cAMP (Deutscher et al., 2006). However, in unknown conditions in which the activity of Crr is likely reduced in *K. pneumoniae*, the activity of EtcABC (or EtcA, as discussed below) is increased to enhance the AC activity to produce cAMP. The increased level of intracellular cAMP leads to an increased level of CRP-cAMP and then activates the transcription of the *lac* and *mrk* operons. Activation of the latter operon results in the promotion of type 3 fimbriae synthesis, biofilm formation, and adhesion to cells in *K. pneumoniae* (Figure 6). However, how does CRP activate the transcription of *mrk* operon? Previous studies showed that MrkH positively regulates *mrkHI* bicistronic and *mrkABCDF* operon by direct binding on the upstream region of the target genes in the presence of c-di-GMP (Wilksch et al., 2011; Tan et al., 2015). In this study, the transcription of *mrkH* is reduced significantly in *crp* mutant, compared to wild type (Supplementary Figures S3D–F). We speculate that CRP positively regulates the transcription of *mrkH*. Then, MrkH activates the *mrkABCDF* operon to produce type 3 fimbriae. We are constructing the *mrkH* mutant overexpressing CRP and *crp* mutant overexpressing MrkH to confirm whether regulation of *mrkABCDF* by CRP can be independent on MrkH. However, the role of CRP binding to the upstream of *mrkA* in *K. pneumoniae* STU1 is unclear in the present study. Perhaps, binding of CRP to this site affects the neighbor region in the opposite direction of *mrkA*.

Luo et al. (2017) reported that the *KP1-4563* gene located upstream of the *mrk* cluster in *K. pneumoniae* NTUH-K2044 encodes a hypothetical protein with a putative conserved domain, DUF1471, with an unknown function. The authors reported that the CRP binding site located upstream of *KP1-4563* and CRP negatively regulated *KP1-4563*. Compared to the wild-type strain, the *KP1-4563*-deleted mutant strain exhibited increased activity in the MRHA, mannan-binding assay and bacterial adhesion assay. Therefore, Luo et al. (2017) suggested that *KP1-4563* negatively regulates the function of type 3 fimbriae. However, these three assays in the report by Luo et al. (2017) did not allow direct observation of type 3 fimbriae synthesis such as Western blotting using antibody specific to type 3 fimbriae. Some other factors in *K. pneumoniae* may affect the results of MRHA (Stahlhut et al., 2012), mannan-binding assay (Madison et al., 1994), and bacterial adhesion assay (Chung, 2016; Horng et al., 2018). Therefore, the regulation of MRHA, mannan-binding assay and bacterial adhesion assay by *KP1-4563* depending on type 3 fimbriae or not is unclear. However, we found KPN03280, located upstream of *mrkA* in *K. pneumoniae* MGH78578, is homologous to *KP1-4563* in *K. pneumoniae* NTUH-K2044. The putative CRP binding site was located upstream of KPN03280 in *K. pneumoniae* MGH78578. Although the upstream of *mrkA* in *K. pneumoniae* STU1 is not clarified, open reading frame homologous to *KP1-4563* is possibly located upstream of *mrkA* in *K. pneumoniae* STU1. Thus, the effect of CRP on type 3 fimbriae should be further investigated to clarify whether the mechanism operates through indirect control via *KP1-4563* homolog.

Overexpression of *etcABC* increased MrkA expression, compared to the vector control (Figure 3). We found that the strain overexpressing *etcA* showed greater MrkA production than the vector control but less MrkA expression than the strain overexpressing *etcABC* (data not shown). Therefore, here, we studied the effects of *etcABC* overexpression instead of *etcA* overexpression in *K. pneumoniae*. Previous studies showed that phosphorylation state of EIIA affects its activity (Deutscher et al., 2006; Deuschle et al., 2015). Therefore, we hypothesize that the different results from *etcABC* overexpression and *etcA* overexpression were due to the phosphorylation levels of EtcA in bacteria. However, we could not confirm the phosphorylation state of EtcA in this study. We previously found that KPN00353 is in the fructose-mannitol EIIA (EII^Fru^ and EIIA^Mtl^) family, a subfamily of the glucose-fructose-lactose PTS superfamily. There is 98% identity between EtcA and KPN00353 amino acid sequence. Crr is an EIIA in the glucose family, another subfamily of the glucose-fructose-lactose PTS superfamily. The identity between KPN00353 and Crr is not very high (Jeng et al., 2017). Therefore, the interaction of EtcA with AC and the phosphorylation state of EtcA need further study.

Because type 3 fimbriae play an important role in *K. pneumoniae* biofilm formation, several transcription factors have been reported to be involved in regulating type 3 fimbriae expression. These transcription factors include the histone-like nucleoid-structuring protein (H-NS); MrkHI, which depends on c-di-GMP; IscR, which also acts as a crucial transcriptional regulator to control iron–sulfur (Fe–S) cluster biosynthesis in bacteria; and OmpR, which is a response regulator in the OmpR/EnvZ two-component system, which senses osmotic conditions (Tan et al., 2015; Ares et al., 2016; Lin et al., 2017, 2018). We found that overexpression of EtcABC, EII components of a PTS, positively regulate type 3 fimbriae synthesis in *K. pneumoniae* via CRP. To our knowledge, EtcABC (homologous to KPN00353-KPN00352-KPN00351) is a unique EII complex of PTS in *K. pneumoniae* strains and is not found in other bacterial strains (Jeng et al., 2017). Therefore, EtcABC may play a unique role in *K. pneumoniae* pathogenesis. One limitation of this study is that the role of EtcABC in regulating type 3 fimbrial synthesis under various physiologic conditions is unclear. For example, we found that an *etcABC* mutant had similar expression of type 3 fimbriae when grown in LB media compared to wild type. There may, however, be environmental conditions in which EtcABC is more highly expressed and therefore does affect type 3 fimbriae expression as demonstrated by our experiments utilizing EtcABC overexpression in trans.

## Conclusion

In conclusion, we showed that the CRP-cAMP signaling pathway positively regulated the *lac* operon and type 3 fimbriae synthesis by activating *mrkABCDF* in at least three *K. pneumoniae* isolates. Moreover, cAMP played a critical role in *lac* operon activation. The glucose-specific EIIA, Crr, positively regulated the *lac* operon via AC, which produces cAMP. Moreover, the putative EII complex EtcABC overexpression compensated for the role of Crr in *lac* operon activation and positively regulated type 3 fimbriae synthesis by increasing the transcription level of *mrkABCDF* in *K. pneumoniae* via CRP-cAMP. Thus, we concluded that *K. pneumoniae* modulated biofilm formation via the PTS and the CRP-cAMP signaling pathway.

## Author Contributions

NP performed most of the experiments and wrote and revised the manuscript. Y-TH analyzed the data and wrote and revised the manuscript. S-WC constructed the suicide plasmid and *crp* mutant. W-TC purified the CRP and performed the EMSA. P-CS conceived and designed the study, performed and supervised the experiments, analyzed the data, and wrote and revised the manuscript.

## Conflict of Interest Statement

The authors declare that the research was conducted in the absence of any commercial or financial relationships that could be construed as a potential conflict of interest.

## References

[B1] Allen B. L. Gerlach G. F. Clegg S. (1991). Nucleotide sequence and functions of mrk determinants necessary for expression of type 3 fimbriae in *Klebsiella pneumoniae*. *J. Bacteriol.* 173 916–920. 10.1128/jb.173.2.916-920.1991 1670938 PMC207091

[B2] Ares M. A. Fernandez-Vazquez J. L. Rosales-Reyes R. Jarillo-Quijada M. D. von Bargen K. Torres J. (2016). H-NS nucleoid protein controls virulence features of *Klebsiella pneumoniae* by regulating the expression of type 3 Pili and the Capsule Polysaccharide. *Front. Cell. Infect. Microbiol.* 6:13. 10.3389/fcimb.2016.00013 26904512 PMC4746245

[B3] Baldauf S. L. Cardani M. A. Bender R. A. (1988). Regulation of the galactose-inducible lac operon and the histidine utilization operons in pts mutants of Klebsiella aerogenes. *J. Bacteriol.* 170 5588–5593. 10.1128/jb.170.12.5588-5593.1988 3142852 PMC211655

[B4] Chang A. C. Cohen S. N. (1978). Construction and characterization of amplifiable multicopy DNA cloning vehicles derived from the P15A cryptic miniplasmid. *J. Bacteriol.* 134 1141–1156. 149110 10.1128/jb.134.3.1141-1156.1978 PMC222365

[B5] Chung P. Y. (2016). The emerging problems of *Klebsiella pneumoniae* infections: carbapenem resistance and biofilm formation. *FEMS Microbiol. Lett.* 363:fnw219. 10.1093/femsle/fnw219 27664057

[B6] Clegg S. Murphy C. N. (2016). Epidemiology and virulence of *Klebsiella pneumoniae*. *Microbiol. Spectr.* 4:UTI-0005-2012. 10.1128/microbiolspec.UTI-0005-2012 26999397

[B7] de Lorenzo V. Herrero M. Jakubzik U. Timmis K. N. (1990). Mini-Tn5 transposon derivatives for insertion mutagenesis, promoter probing, and chromosomal insertion of cloned DNA in gram-negative eubacteria. *J. Bacteriol.* 172 6568–6572. 10.1128/jb.172.11.6568-6572.1990 2172217 PMC526846

[B8] Deuschle M. Limbrunner S. Rother D. Wahler S. Chavarria M. de Lorenzo V. (2015). Interplay of the PtsN (EIIA(Ntr)) protein of *Pseudomonas* putida with its target sensor kinase KdpD. *Environ. Microbiol. Rep.* 7 899–907. 10.1111/1758-2229.12323 26224366

[B9] Deutscher J. Ake F. M. Derkaoui M. Zebre A. C. Cao T. N. Bouraoui H. (2014). The bacterial phosphoenolpyruvate:carbohydrate phosphotransferase system: regulation by protein phosphorylation and phosphorylation-dependent protein-protein interactions. *Microbiol. Mol. Biol. Rev.* 78 231–256. 10.1128/MMBR.00001-14 24847021 PMC4054256

[B10] Deutscher J. Francke C. Postma P. W. (2006). How phosphotransferase system-related protein phosphorylation regulates carbohydrate metabolism in bacteria. *Microbiol. Mol. Biol. Rev.* 70 939–1031. 10.1128/MMBR.00024-26 17158705 PMC1698508

[B11] Feucht B. U. Saier M. H. Jr. (1980). Fine control of adenylate cyclase by the phosphoenolpyruvate:sugar phosphotransferase systems in *Escherichia coli* and *Salmonella* typhimurium. *J. Bacteriol.* 141 603–610. 6245052 10.1128/jb.141.2.603-610.1980 PMC293665

[B12] Gosset G. Zhang Z. Nayyar S. Cuevas W. A. Saier M. H. Jr. (2004). Transcriptome analysis of Crp-dependent catabolite control of gene expression in *Escherichia coli*. *J. Bacteriol.* 186 3516–3524. 10.1128/JB.186.11.3516-3524.2004 15150239 PMC415760

[B13] Green J. Stapleton M. R. Smith L. J. Artymiuk P. J. Kahramanoglou C. Hunt D. M. (2014). Cyclic-AMP and bacterial cyclic-AMP receptor proteins revisited: adaptation for different ecological niches. *Curr. Opin. Microbiol.* 18 1–7. 10.1016/j.mib.2014.01.003 24509484 PMC4005916

[B14] Gunasekera A. Ebright Y. W. Ebright R. H. (1992). DNA sequence determinants for binding of the *Escherichia coli* catabolite gene activator protein. *J. Biol. Chem.* 267 14713–14720. 1321815

[B15] Guzman L. M. Belin D. Carson M. J. Beckwith J. (1995). Tight regulation, modulation, and high-level expression by vectors containing the arabinose PBAD promoter. *J. Bacteriol.* 177 4121–4130. 10.1128/jb.177.14.4121-4130.1995 7608087 PMC177145

[B16] Harman J. G. (2001). Allosteric regulation of the cAMP receptor protein. *Biochim. Biophys. Acta* 1547 1–17. 10.1016/s0167-4838(01)00187-x 11343786

[B17] Horng Y. T. Wang C. J. Chung W. T. Chao H. J. Chen Y. Y. Soo P. C. (2018). Phosphoenolpyruvate phosphotransferase system components positively regulate Klebsiella biofilm formation. *J. Microbiol. Immunol. Infect.* 51 174–183. 10.1016/j.jmii.2017.01.007 28716362

[B18] Hornick D. B. Allen B. L. Horn M. A. Clegg S. (1992). Adherence to respiratory epithelia by recombinant *Escherichia coli* expressing *Klebsiella pneumoniae* type 3 fimbrial gene products. *Infect. Immun.* 60 1577–1588. 1312518 10.1128/iai.60.4.1577-1588.1992 PMC257033

[B19] Ishizuka H. Hanamura A. Inada T. Aiba H. (1994). Mechanism of the down-regulation of cAMP receptor protein by glucose in *Escherichia coli*: role of autoregulation of the crp gene. *EMBO J.* 13 3077–3082. 10.1002/j.1460-2075.1994.tb06606.x 7518773 PMC395198

[B20] Jagnow J. Clegg S. (2003). *Klebsiella pneumoniae* MrkD-mediated biofilm formation on extracellular matrix- and collagen-coated surfaces. *Microbiology* 149(Pt 9), 2397–2405. 10.1099/mic.0.26434-26430 12949165

[B21] Jeng W. Y. Panjaitan N. S. D. Horng Y. T. Chung W. T. Chien C. C. Soo P. C. (2017). The Negative Effects of KPN00353 on Glycerol Kinase and Microaerobic 1,3-Propanediol Production in *Klebsiella pneumoniae*. *Front. Microbiol.* 8:2441. 10.3389/fmicb.2017.02441 29375490 PMC5770620

[B22] Johnson J. G. Murphy C. N. Sippy J. Johnson T. J. Clegg S. (2011). Type 3 fimbriae and biofilm formation are regulated by the transcriptional regulators MrkHI in *Klebsiella pneumoniae*. *J. Bacteriol.* 193 3453–3460. 10.1128/JB.00286-211 21571997 PMC3133326

[B23] Kolpa M. Walaszek M. Gniadek A. Wolak Z. Dobros W. (2018). Incidence, microbiological profile and risk factors of healthcare-associated infections in intensive care units: a 10 year observation in a Provincial hospital in Southern Poland. *Int. J. Environ. Res. Public Health* 15:E112. 10.3390/ijerph15010112 29324651 PMC5800211

[B24] Lin C. T. Lin T. H. Wu C. C. Wan L. Huang C. F. Peng H. L. (2016). CRP-Cyclic AMP Regulates the Expression of Type 3 Fimbriae via Cyclic di-GMP in *Klebsiella pneumoniae*. *PLoS One* 11:e0162884. 10.1371/journal.pone.0162884 27631471 PMC5025149

[B25] Lin T. H. Chen Y. Kuo J. T. Lai Y. C. Wu C. C. Huang C. F. (2018). Phosphorylated OmpR Is Required for Type 3 Fimbriae Expression in *Klebsiella pneumoniae* Under Hypertonic Conditions. *Front. Microbiol.* 9:2405. 10.3389/fmicb.2018.02405 30369914 PMC6194325

[B26] Lin T. H. Tseng C. Y. Lai Y. C. Wu C. C. Huang C. F. Lin C. T. (2017). IscR Regulation of Type 3 Fimbriae Expression in *Klebsiella pneumoniae* CG43. *Front. Microbiol.* 8:1984. 10.3389/fmicb.2017.01984 29085346 PMC5650617

[B27] Lin Y. S. Green M. R. (1989). Similarities between prokaryotic and eukaryotic cyclic AMP-responsive promoter elements. *Nature* 340 656–659. 10.1038/340656a0 2549425

[B28] Luo M. Yang S. Li X. Liu P. Xue J. Zhou X. (2017). The KP1_4563 gene is regulated by the cAMP receptor protein and controls type 3 fimbrial function in *Klebsiella pneumoniae* NTUH-K2044. *PLoS One* 12:e0180666. 10.1371/journal.pone.0180666 28732013 PMC5521740

[B29] Madison B. Ofek I. Clegg S. Abraham S. N. (1994). Type 1 fimbrial shafts of *Escherichia coli* and *Klebsiella pneumoniae* influence sugar-binding specificities of their FimH adhesins. *Infect. Immun.* 62 843–848. 7906676 10.1128/iai.62.3.843-848.1994 PMC186191

[B30] March C. Moranta D. Regueiro V. Llobet E. Tomas A. Garmendia J. (2011). *Klebsiella pneumoniae* outer membrane protein A is required to prevent the activation of airway epithelial cells. *J. Biol. Chem.* 286 9956–9967. 10.1074/jbc.M110.181008 21278256 PMC3060550

[B31] Narang A. (2009). Quantitative effect and regulatory function of cyclic adenosine 5′-phosphate in *Escherichia coli*. *J. Biosci.* 34 445–463. 10.1007/s12038-009-0051-1 19805906

[B32] Novichkov P. S. Laikova O. N. Novichkova E. S. Gelfand M. S. Arkin A. P. Dubchak I. (2010). RegPrecise: a database of curated genomic inferences of transcriptional regulatory interactions in prokaryotes. *Nucleic Acids Res.* 38 D111–D118. 10.1093/nar/gkp894 19884135 PMC2808921

[B33] Ong C. L. Beatson S. A. Totsika M. Forestier C. McEwan A. G. Schembri M. A. (2010). Molecular analysis of type 3 fimbrial genes from *Escherichia coli*, Klebsiella and Citrobacter species. *BMC Microbiol.* 10:183. 10.1186/1471-2180-10-183 20576143 PMC2900259

[B34] O’Toole G. A. Kolter R. (1998). Initiation of biofilm formation in *Pseudomonas* fluorescens WCS365 proceeds via multiple, pathways convergent signalling: a genetic analysis. *Mol. Microbiol.* 28 449–461. 10.1046/j.1365-2958.1998.00797.x 9632250

[B35] Ou Q. Fan J. Duan D. Xu L. Wang J. Zhou D. (2017). Involvement of cAMP receptor protein in biofilm formation, fimbria production, capsular polysaccharide biosynthesis and lethality in mouse of *Klebsiella pneumoniae* serotype K1 causing pyogenic liver abscess. *J. Med. Microbiol.* 66 1–7. 10.1099/jmm.0.000391 27902401

[B36] Percival S. L. Suleman L. Vuotto C. Donelli G. (2015). Healthcare-associated infections, medical devices and biofilms: risk, tolerance and control. *J. Med. Microbiol.* 64(Pt 4), 323–334. 10.1099/jmm.0.000032 25670813

[B37] Reid G. Charbonneau-Smith R. Lam D. Kang Y. S. Lacerte M. Hayes K. C. (1992). Bacterial biofilm formation in the urinary bladder of spinal cord injured patients. *Paraplegia* 30 711–717. 10.1038/sc.1992.138 1448299

[B38] Rosen D. A. Pinkner J. S. Walker J. N. Elam J. S. Jones J. M. Hultgren S. J. (2008). Molecular variations in *Klebsiella pneumoniae* and *Escherichia coli* FimH affect function and pathogenesis in the urinary tract. *Infect. Immun.* 76 3346–3356. 10.1128/IAI.00340-348 18474655 PMC2446687

[B39] Schafer A. Tauch A. Jager W. Kalinowski J. Thierbach G. Puhler A. (1994). Small mobilizable multi-purpose cloning vectors derived from the *Escherichia coli* plasmids pK18 and pK19: selection of defined deletions in the chromosome of Corynebacterium glutamicum. *Gene* 145 69–73. 10.1016/0378-1119(94)90324-7 8045426

[B40] Schroll C. Barken K. B. Krogfelt K. A. Struve C. (2010). Role of type 1 and type 3 fimbriae in *Klebsiella pneumoniae* biofilm formation. *BMC Microbiol.* 10:179. 10.1186/1471-2180-10-179 20573190 PMC2911432

[B41] Schumacher M. A. Zeng W. (2016). Structures of the activator of K. *pneumonia biofilm formation*, MrkH, indicates PilZ domains involved in c-di-GMP and DNA binding. *Proc Natl Acad Sci U.S.A.* 113 10067–10072. 10.1073/pnas.1607503113 27551088 PMC5018759

[B42] Sebghati T. A. Korhonen T. K. Hornick D. B. Clegg S. (1998). Characterization of the type 3 fimbrial adhesins of Klebsiella strains. *Infect. Immun.* 66 2887–2894. 9596764 10.1128/iai.66.6.2887-2894.1998 PMC108286

[B43] Simon R. Priefer U. Pühler A. (1983). A broad host range mobilization system for in vivo genetic engineering: transposon mutagenesis in gram negative bacteria. *Bio/Technology* 1 784–791. 10.1038/nbt1183-784

[B44] Singhai M. Malik A. Shahid M. Malik M. A. Goyal R. (2012). A study on device-related infections with special reference to biofilm production and antibiotic resistance. *J. Glob. Infect. Dis.* 4 193–198. 10.4103/0974-777X.103896 23326076 PMC3543538

[B45] Soo P. C. Horng Y. T. Chang Y. L. Tsai W. W. Jeng W. Y. Lu C. C. (2014). ManA is regulated by RssAB signaling and promotes motility in *Serratia marcescens*. *Res. Microbiol.* 165 21–29. 10.1016/j.resmic.2013.10.005 24161484

[B46] Soo P. C. Horng Y. T. Lai M. J. Wei J. R. Hsieh S. C. Chang Y. L. (2007). Pirin regulates pyruvate catabolism by interacting with the pyruvate dehydrogenase E1 subunit and modulating pyruvate dehydrogenase activity. *J. Bacteriol.* 189 109–118. 10.1128/JB.00710-716 16980458 PMC1797226

[B47] Soo P. C. Horng Y. T. Wei J. R. Shu J. C. Lu C. C. Lai H. C. (2008). Regulation of swarming motility and flhDC(Sm) expression by RssAB signaling in *Serratia marcescens*. *J. Bacteriol.* 190 2496–2504. 10.1128/JB.01670-1677 18223092 PMC2293207

[B48] Stahlhut S. G. Struve C. Krogfelt K. A. (2012). *Klebsiella pneumoniae* type 3 fimbriae agglutinate yeast in a mannose-resistant manner. *J. Med. Microbiol.* 61(Pt 3), 317–322. 10.1099/jmm.0.036350-36350 22016558

[B49] Struve C. Bojer M. Krogfelt K. A. (2008). Characterization of *Klebsiella pneumoniae* type 1 fimbriae by detection of phase variation during colonization and infection and impact on virulence. *Infect. Immun.* 76 4055–4065. 10.1128/IAI.00494-498 18559432 PMC2519443

[B50] Takahashi M. Blazy B. Baudras A. (1980). An equilibrium study of the cooperative binding of adenosine cyclic 3′,5′-monophosphate and guanosine cyclic 3′,5′-monophosphate to the adenosine cyclic 3′,5′-monophosphate receptor protein from *Escherichia coli*. *Biochemistry* 19 5124–5130. 10.1021/bi00563a029 6257276

[B51] Takahashi M. Blazy B. Baudras A. Hillen W. (1989). Ligand-modulated binding of a gene regulatory protein to DNA. Quantitative analysis of cyclic-AMP induced binding of CRP from *Escherichia coli* to non-specific and specific DNA targets. *J. Mol. Biol.* 207 783–796. 2547972 10.1016/0022-2836(89)90244-1

[B52] Tan J. W. Wilksch J. J. Hocking D. M. Wang N. Srikhanta Y. N. Tauschek M. (2015). Positive autoregulation of mrkHI by the cyclic di-GMP-dependent MrkH protein in the biofilm regulatory circuit of *Klebsiella pneumoniae*. *J. Bacteriol.* 197 1659–1667. 10.1128/JB.02615-2614 25733612 PMC4403657

[B53] Tarkkanen A. M. Virkola R. Clegg S. Korhonen T. K. (1997). Binding of the type 3 fimbriae of *Klebsiella pneumoniae* to human endothelial and urinary bladder cells. *Infect. Immun.* 65 1546–1549. 9119502 10.1128/iai.65.4.1546-1549.1997 PMC175168

[B54] Vuotto C. Longo F. Balice M. P. Donelli G. Varaldo P. E. (2014). Antibiotic Resistance Related to Biofilm Formation in *Klebsiella pneumoniae*. *Pathogens* 3 743–758. 10.3390/pathogens3030743 25438022 PMC4243439

[B55] Wang H. Wilksch J. J. Chen L. Tan J. W. Strugnell R. A. Gee M. L. (2017). Influence of fimbriae on bacterial adhesion and viscoelasticity and correlations of the two properties with biofilm formation. *Langmuir* 33 100–106. 10.1021/acs.langmuir.6b03764 27959542

[B56] Wilksch J. J. Yang J. Clements A. Gabbe J. L. Short K. R. Cao H. (2011). MrkH, a novel c-di-GMP-dependent transcriptional activator, controls *Klebsiella pneumoniae* biofilm formation by regulating type 3 fimbriae expression. *PLoS Pathog.* 7:e1002204. 10.1371/journal.ppat.1002204 21901098 PMC3161979

[B57] Zheng D. Constantinidou C. Hobman J. L. Minchin S. D. (2004). Identification of the CRP regulon using in vitro and in vivo transcriptional profiling. *Nucleic. Acids Res.* 32 5874–5893. 10.1093/nar/gkh908 15520470 PMC528793

