# Supplementary material for: EtcABC, a Putative EII Complex, Regulates Type 3 Fimbriae via CRP-cAMP Signaling in Klebsiella pneumoniae
Source: Front Microbiol. 2019 Jul 9;10:1558. doi: 10.3389/fmicb.2019.01558 (PMC6629953; doi:10.3389/fmicb.2019.01558)
Supplement: Supplementary file 1 [file Data_Sheet_1.PDF]

Supplementary Table S1. Oligonucleotide primers used in this study.

Primer	Sequence (5'→3')	Target/purpose
RNA 16S-1	ATGACCAGCCACACTGGAAC	16S rRNA/qRT-PCR
RNA 16S-2	CTTCCTCCCCGCTGAAAGTA	16S rRNA/qRT-PCR
MrkD qPCR FP	TGGTAGCGGTAGTGCTTGTG	*mrkD* mRNA/ qRT-PCR
MrkD qPCR RP	GCTGAGCGGGAAAAATATGA	*mrkD* mRNA/ qRT-PCR
MrkA qPCR FP	GCGGTAGCGTTGTCAGTAGA	*mrkA* mRNA/ qRT-PCR
MrkA qPCR RP	GCTGCTGATGGCACTAAACA	*mrkA* mRNA/ qRT-PCR
FimA qPCR FP	GGACCGTGCATTTTAAAGGA	*fimA* mRNA/ qRT-PCR
FimA qPCR RP	GGCTACCGTAGTGTCGCAAT	*fimA* mRNA/ qRT-PCR
FimH qPCR FP	CGGTGTGCTGTCGAGTTTT	*fimH* mRNA/ qRT-PCR
FimH qPCR RP	GTTGTTGGTCTGATGCAGGA	*fimH* mRNA/ qRT-PCR
MrkH qPCR FP	GCTACGCTTTTTCATTGCC	*mrkH* mRNA/ qRT-PCR
MrkH qPCR RP	AACCGCCATCTGAAATGTC	*mrkH* mRNA/ qRT-PCR
Con etcCFP	TTATTCATTTTTCTGCCTCG	*etcABC*/overexpression of *etcABC*
Con etcARP	ATTCGCCCTACCTCTCACTTT	*etcABC* /overexpression of *etcABC*
crr up FP	GGCGGCCGCCCCGGGAAATTGAAGCGCTGCGTAGT	upstream of *crr*/ *crr* mutant construction
crr up RP	GGGGATCCGCATCTCGTGGATTAGCAGA	upstream of *crr*/ *crr* mutant construction
crr down FP	GGGGATCCCGGTAATCCGCATCAAGAAG	downstream of *crr*/ *crr* mutant construction
crr down RP	GGGAGCTCCCCGGGACGCCTATCGTCAGCACCT	downstream of *crr*/ *crr* mutant construction
crr FP	CCCGGGGACGAGTTAATGACGCTGGTT	*crr* / *crr* complementation
crr RP	AAGCTTGTGCTATCGACCGGTAACCT	*crr* / *crr* complementation
yhfA' FP	GAATTCCTGATGGAAGGCCGTAAGAT	upstream of *crp*/ *crp* mutant construction
yhfA' RP	AAGCTTGCGCGGTTATCCTCTGTTAT	upstream of *crp*/ *crp* mutant construction
yhfK' FP	AAGCTTTACGCCGTTTTTGTTTTTCC	downstream of *crp*/ *crp* mutant construction
yhfK' RP	GTCGACGTAGATCCAGCCCCATCTGA	downstream of *crp*/ *crp* mutant construction
crp FP	GAATTCAATGGTGCTTGGCAAACCGC	*crp* / *crp* complementation
crp RP	GAATTCTTAACGGGTGCCGTAGACGA	*crp* / *crp* complementation
crp promoter FP	GGGATATCACCCTTCTACCCATTTCACA	*crp* promoter region /P*crp-luxCDABE* reporter construction
crp promoter RP	GGGGATCCTGTGGCAATGAGACAAGAAC	*crp* promoter region /P*crp-luxCDABE* reporter construction
cyaA up FP	GGAATTCCCCGGGTTGCCAGCGGAGTATCAAG	upstream of *cyaA* / *cyaA* mutant construction
cyaA up RP	GGGATCCTCGATCAGGAAGAAGCTGACT	upstream of *cyaA* / *cyaA* mutant construction
cyaA down FP	GGGATCCATCCGGGAAACACGTCATTA	downstream of *cyaA* / *cyaA* mutant construction
cyaA down RP	GGAGCTCCCCGGGCAAGGCCATGCATTTTAGAGA	downstream of *cyaA* / *cyaA* mutant construction
cyaA FP	CCCGGGAAATGTACCTCTATATTGAGACACTGA	*cyaA* / *cyaA* complementation
cyaA RP	CTACGAGTAGCGCTGCTGCA	*cyaA* / *cyaA* complementation

etcC up FP	GAATTCCCCGGGATTTAACGAGTGGTGGACGA	downstream of *etcC* / *etcABC* mutant construction
etcC up RP	GGATCCAATAAGCGAGGTCATTTTTCC	downstream of *etcC* / *etcABC* mutant construction
etcA down FP	GGATCCCAGCTCCTGAACGTATTCCTG	upstream of *etcA / etcABC* mutant construction
etcA down RP	CCCGGGGAAGTGGTTGGCGTTTCCT	upstream of *etcA / etcABC* mutant construction
MrkA FP	GTCGACTTACTGGTAAGTAATTTCGT	*mrkA*/ His tagged MrkA construction
MrkA RP	GGATCCAATGGCAACCGCGTTTTTT	*mrkA*/ His tagged MrkA construction
FimA FP	GGGGAATTCAATGAAAATCAAAACACTGGC	*fimA*/ His tagged FimA construction
FimA RP	GGGGCGGCCGCTTACTCGTACTGCACTTTGA	*fimA*/ His tagged FimA construction
MrkA EMSA FP	GCCATCGCGATAAGTCTGA	upstream of *mrkA*/EMSA
MrkA EMSA RP	ATGAGCGAAGCTAGCGAAAG	upstream of *mrkA*/EMSA
etcA F EMSA	CGCCGAATATGTCAACGAGA	*etcA* /negative control in EMSA
etcA R EMSA	ATATGACTGTCAGCATCGGC	*etcA* /negative control in EMSA
