# Supplementary material for: EtcABC, a Putative EII Complex, Regulates Type 3 Fimbriae via CRP-cAMP Signaling in Klebsiella pneumoniae
Source: Front Microbiol. 2019 Jul 9;10:1558. doi: 10.3389/fmicb.2019.01558 (PMC6629953; doi:10.3389/fmicb.2019.01558)
Supplement: Supplementary file 2 [file Data_Sheet_2.PDF]

Figure S1

(A)

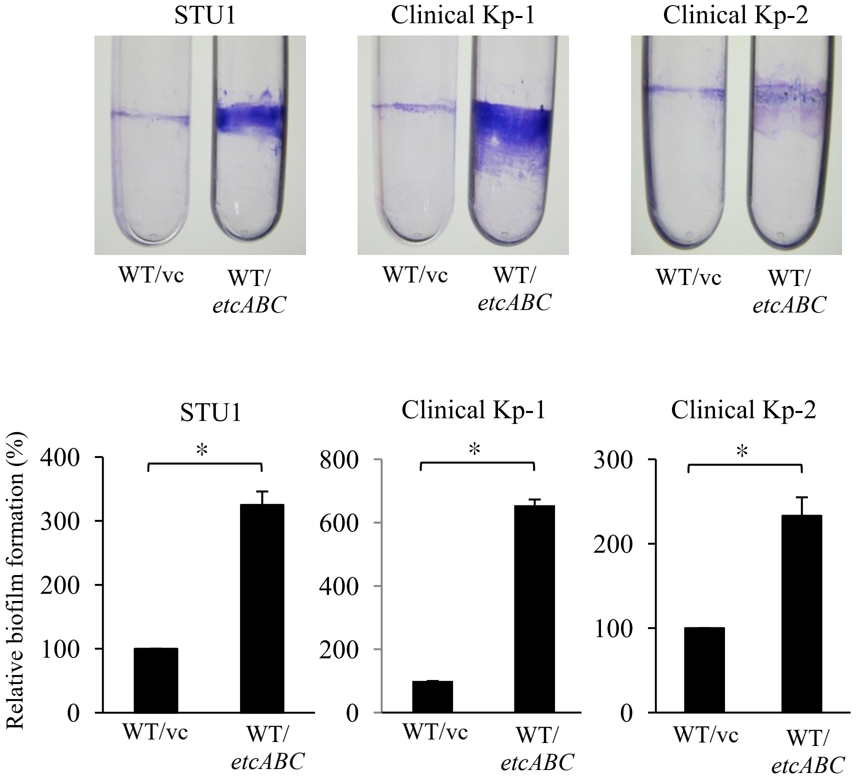

(B)

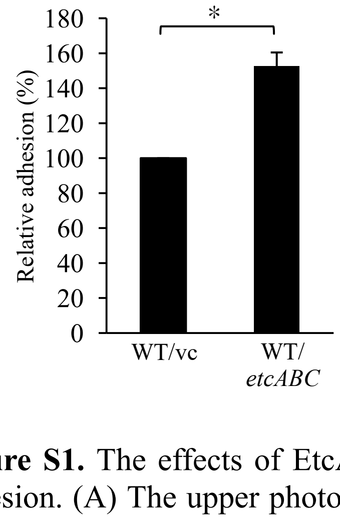

Figure S1. The effects of EtcABC overexpression on bacterial biofilm formation and adhesion. (A) The upper photos showed the bacterial biofilm stained with 0.1% crystal violet. The lower figure showed the biofilm amount of bacteria carrying pBSK::Gm::etcABC (WT/*etcABC*) compared with that of wild-type strains carrying pBSK::Gm (WT/vc). *K. pneumoniae* STU1, Clinical Kp-1 and Clinical Kp-2 are parent strains (WT). (B) The adhesion of *K. pneumoniae* STU1 on the A549 cells. WT/vc: *K. pneumoniae* STU1 carrying pBSK::Gm. WT/*etcABC*: *K. pneumoniae* STU1 carrying pBSK::Gm::etcABC. The amount of WT/*etcABC* adhered on cells was compared to that of WT/vc on cells. In both (A) and (B), the data are presented as the averages ± standard deviations of at least three replicates. An asterisk (*) represents $p < 0.05$ as compared with WT/vc.
