# Supplementary material for: EtcABC, a Putative EII Complex, Regulates Type 3 Fimbriae via CRP-cAMP Signaling in Klebsiella pneumoniae
Source: Front Microbiol. 2019 Jul 9;10:1558. doi: 10.3389/fmicb.2019.01558 (PMC6629953; doi:10.3389/fmicb.2019.01558)
Supplement: Supplementary file 3 [file Data_Sheet_3.PDF]

Figure S2

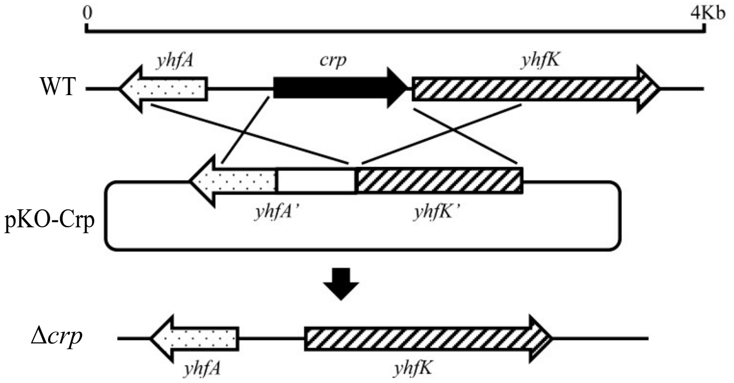

Figure S2. Strategy for constructing the *crp*-deleted mutant strain from *K. pneumoniae* by homologous recombination.
