# Supplementary material for: EtcABC, a Putative EII Complex, Regulates Type 3 Fimbriae via CRP-cAMP Signaling in Klebsiella pneumoniae
Source: Front Microbiol. 2019 Jul 9;10:1558. doi: 10.3389/fmicb.2019.01558 (PMC6629953; doi:10.3389/fmicb.2019.01558)
Supplement: Supplementary file 4 [file Data_Sheet_4.PDF]

Figure S3

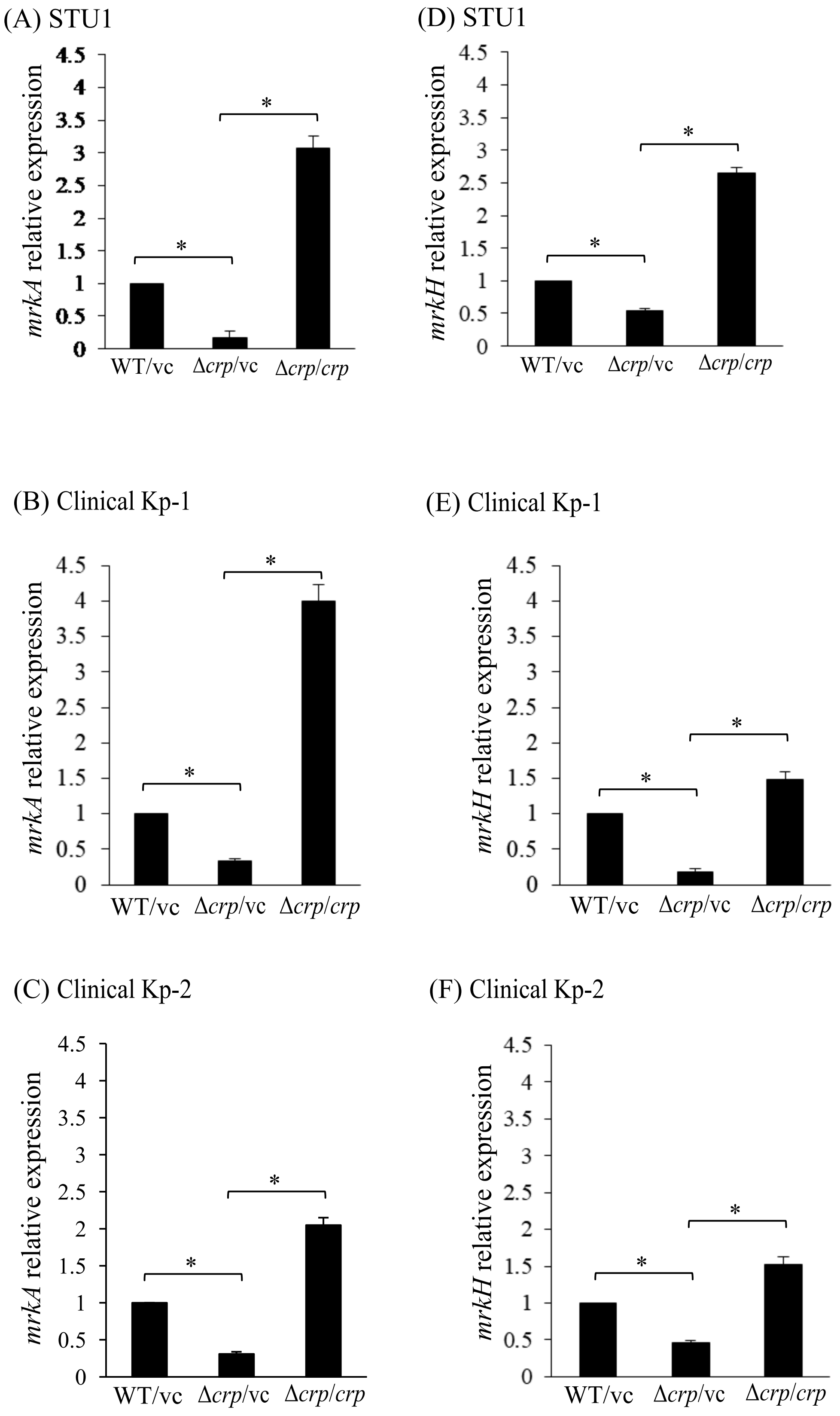

(A) STU1
mrkA relative expression
4.5
4
3.5
3
2.5
2
1.5
1
0.5
0
*
*
WT/vc
Δcrp/vc
Δcrp/crp
(D) STU1
mrkH relative expression
(B) Clinical Kp-1
(E) Clinical Kp-1
(C) Clinical Kp-2
(F) Clinical Kp-2

Figure S3. The effects of *crp* deletion on transcription of *mrkA* and *mrkH* analyzed by RT-qPCR. WT: *K. pneumoniae* (STU1, Clinical Kp-1 or Clinical Kp-2) wild-type strains. Δ*crp*: *K. pneumoniae crp* mutant. vc: bacteria carrying pBAD33 as vector control. Δ*crp*/*crp*: *crp* mutant carrying pBAD33::crp as complement strain. The 16S rRNA gene was used as the reference. Relative gene expressions means transcriptional level of *mrkA* (A, B and C) or *mrkH* (D, E and F) in Δ*crp*/vc or Δ*crp/crp* compared to those in their parent strains carrying vector, WT/vc. The presented results are the means ± standard deviations of three replicates. An asterisk (*) represents $p < 0.05$ as Δ*crp*/vc compared with WT/vc or Δ*crp*/*crp* compared with Δ*crp*/vc.
