# Supplementary material for: EtcABC, a Putative EII Complex, Regulates Type 3 Fimbriae via CRP-cAMP Signaling in Klebsiella pneumoniae
Source: Front Microbiol. 2019 Jul 9;10:1558. doi: 10.3389/fmicb.2019.01558 (PMC6629953; doi:10.3389/fmicb.2019.01558)
Supplement: Supplementary file 5 [file Data_Sheet_5.PDF]

Figure S4

(A)

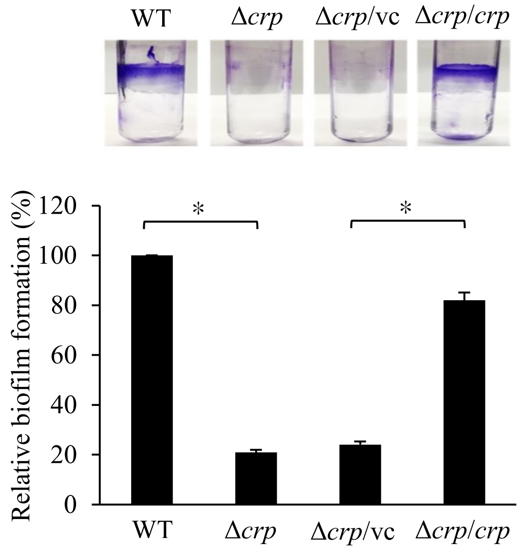

(B)

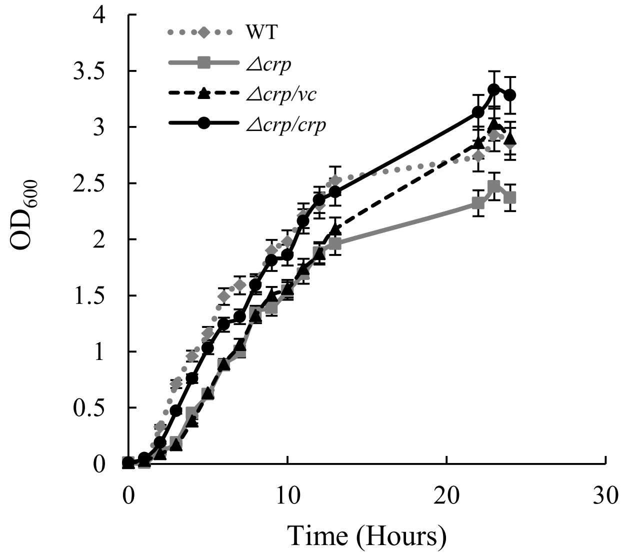

(C)

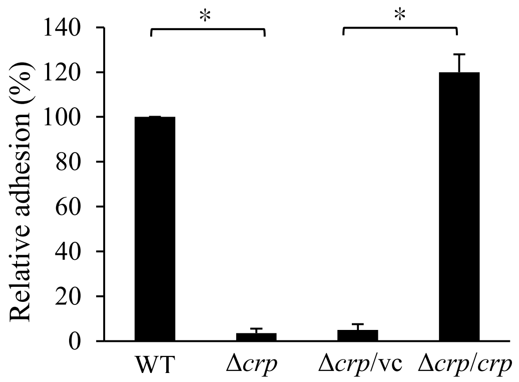

Figure S4. The effects of *crp* deletion on bacterial biofilm formation, growth and adhesion. (A) The upper photos showed the bacterial biofilm stained with 0.1% crystal violet. The lower figure showed the quantification of biofilm from bacteria. (B) The bacterial growth in LB broth was measured by spectrophotometry at 600 nm. (C) The adhesion of *K. pneumoniae* on the A549 cells. In (A) (B) and (C), WT: *K. pneumoniae* STU1. Δ*crp*: *K. pneumoniae* STU1 *crp* mutant. Δ*crp*/*vc*: *crp* mutant carrying pBAD33 as vector control. Δ*crp*/*crp*: *crp* mutant carrying pBAD33::crp as complement strain. The amount of biofilm (A) and adhered bacteria on cells (C) were compared to that of WT respectively. The presented results in (A), (B) and (C) are the means ± standard deviations of three replicates. An asterisk (*) represents $p <0.05$ as compared with WT.
