# Supplementary material for: EtcABC, a Putative EII Complex, Regulates Type 3 Fimbriae via CRP-cAMP Signaling in Klebsiella pneumoniae
Source: Front Microbiol. 2019 Jul 9;10:1558. doi: 10.3389/fmicb.2019.01558 (PMC6629953; doi:10.3389/fmicb.2019.01558)
Supplement: Supplementary file 6 [file Data_Sheet_6.pdf]

Figure S5

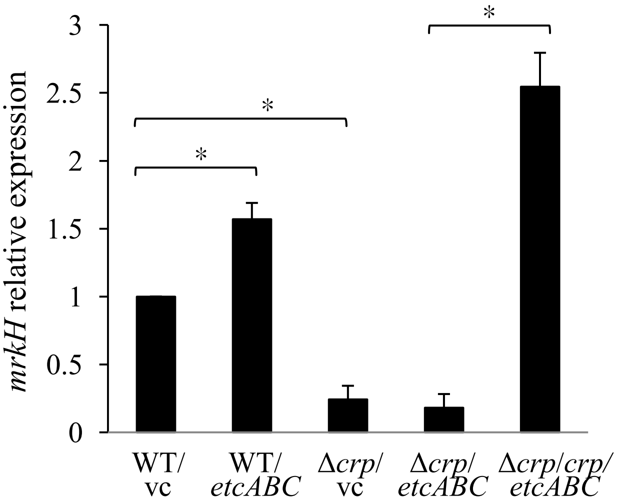

Figure S5. RT-qPCR analysis of *mrkH* transcription in each strain grown in LB. The 16S rRNA gene was used as the reference. The transcriptional level of *mrkH* in each strain was compare to WT/vc. The presented results are the means ± standard deviations of three replicates. An asterisk (*) represents $p < 0.05$ as compared with WT/vc or Δ*crp*/*crp*/*etcABC* compared with Δ*crp*/*etcABC*. WT/vc: *K. pneumoniae* STU1 carrying pBSK-Gm as the vector control. WT/*etcABC*: *K. pneumoniae* STU1 carrying pBSK::Gm::etcABC to overexpress *etcABC*. Δ*crp*/vc: *K. pneumoniae* STU1 *crp* mutant carrying pBSK-Gm as the vector control. Δ*crp/etcABC*: *K. pneumoniae* STU1 *crp* mutant carrying pBSK::Gm::etcABC to overexpress *etcABC*. Δ*crp/crp*/*etcABC*: *K. pneumoniae* STU1 *crp* mutant carrying pBAD33::crp and pBSK::Gm::etcABC. The phenotype of the *crp* mutant carrying the two plasmids, pBAD33 and pBSK::Gm::etcABC, was the same as that of the *crp* mutant carrying one plasmid, pBSK::Gm::etcABC.
