# Supplementary material for: EtcABC, a Putative EII Complex, Regulates Type 3 Fimbriae via CRP-cAMP Signaling in Klebsiella pneumoniae
Source: Front Microbiol. 2019 Jul 9;10:1558. doi: 10.3389/fmicb.2019.01558 (PMC6629953; doi:10.3389/fmicb.2019.01558)
Supplement: Supplementary file 7 [file Data_Sheet_7.PDF]

Figure S6

(A)

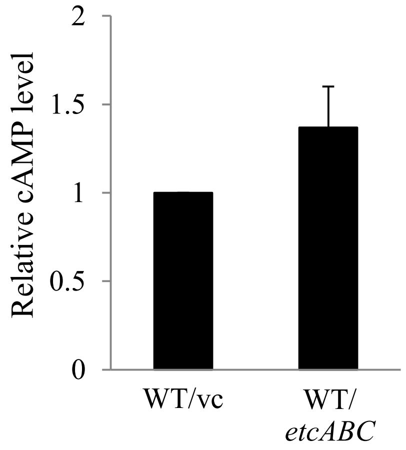

(B)

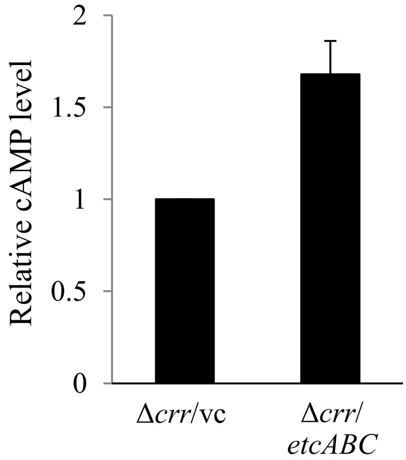

Figure S6. The effects of *etcABC* on cAMP production in wild type (A) and *crr* mutant (B). The intracellular cAMP was quantified by ELISA. WT/vc: *K. pneumoniae* STU1 wild-type carrying pBSK-Gm as the vector control. WT/*etcABC*: wild-type carrying pBSK::Gm::etcABC to overexpress *etcABC*. Δ*crr*/vc: *crr* mutant carrying pBSK-Gm as the vector control. Δ*crr*/*etcABC*: *crr* mutant carrying pBSK::Gm::etcABC to overexpress *etcABC*. Relative cAMP level means the amount of cAMP in bacteria overexpressing *etcABC* compared to those in vector control.
