# Supplementary material for: EtcABC, a Putative EII Complex, Regulates Type 3 Fimbriae via CRP-cAMP Signaling in Klebsiella pneumoniae
Source: Front Microbiol. 2019 Jul 9;10:1558. doi: 10.3389/fmicb.2019.01558 (PMC6629953; doi:10.3389/fmicb.2019.01558)
Supplement: Supplementary file 8 [file Data_Sheet_8.PDF]

Figure S7

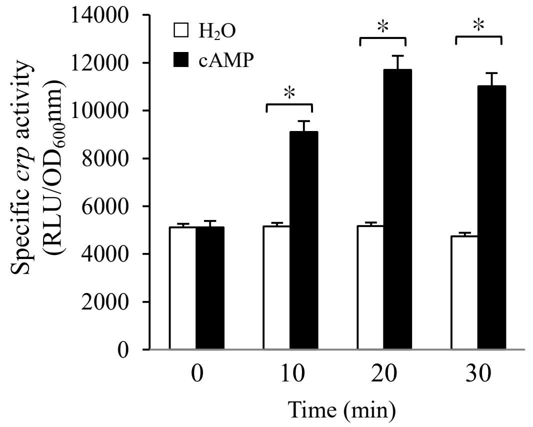

Figure S7. The promoter activity of *crp* was increased by cAMP. After the addition of cAMP (black bar) or water (white bar) to the bacterial culture in LB medium, the luminescence of *K. pneumoniae* STU1 carrying the pPcrp-lux plasmid (the *crp* promoter followed by the *luxCDABE* reporter) was measured at 10, 20 and 30 min. The final concentration of exogenous cAMP in the bacterial culture was 5 mM. An asterisk (*) represents $p < 0.05$ as compared to the bacterial sample added with water.
